# A minimal mathematical model for polarity establishment and centralspindlin-independent cytokinesis

**DOI:** 10.1242/jcs.264093

**Published:** 2025-06-11

**Authors:** Ondrej Maxian, Katrina M. Longhini, Michael Glotzer

**Affiliations:** ^1^ Department of Molecular Genetics and Cell Biology, University of Chicago, Chicago, IL 60637, USA; ^2^ Institute for Biophysical Dynamics, University of Chicago, Chicago, IL 60637, USA

**Keywords:** Cytokinesis, Polarity establishment, Mathematical modelling

## Abstract

Cell polarization and cytokinesis are fundamental processes in organismal development. In the *Caenorhabditis elegans* model system, both processes are partially driven by local inhibition of contractility at the cell poles. This inhibition comes from Aurora A kinase (AIR-1), which is activated on centrosomes and diffuses to the cortex, where it inhibits the guanine nucleotide exchange factor (GEF) ECT-2, attenuating RHO-1 activation and actomyosin-based contractility. Although these biochemical processes have been characterized experimentally, a quantitative understanding of how this circuit drives cortical dynamics in polarization and cytokinesis is still lacking. Here, we constructed a mathematical model to test whether a minimal set of well-characterized, essential elements are necessary and sufficient to explain the spatiotemporal dynamics of AIR-1, ECT-2 and myosin during polarization and cytokinesis of *C. elegans*. We show that robust establishment of polarity can be obtained in response to a weak AIR-1 signal and demonstrate the relevance of rapid ECT-2 exchange and persistent AIR-1 cues during polarization. The model, tuned for polarization, can also predict ECT-2 accumulation during cytokinesis, suggesting a quantitative similarity between the two processes.

## Teaser

**Summary:** Using mathematical models constrained by experimental data, we demonstrate quantitatively that the same cue-driven circuit organizes the cell cortex during polarization and cytokinesis.

## INTRODUCTION

Polarization and cytokinesis are fundamental processes in organismal development and physiology (Dewey et al., 2015). Cell polarization is encoded by asymmetric distributions of protein molecules, which are shaped by local regulation of binding and diffusion, and especially active transport by cortical flows (Munro et al., 2004; Mogilner et al., 2012; Lang and Munro, 2017). Likewise, cytokinesis involves the formation and constriction of the actomyosin ring in the cell mid-plane, a process driven by a balance of contractility, flow and membrane mechanics (White and Borisy, 1983; White, 1985; Glotzer, 2005). The two processes can be biochemically and mechanically connected, as cell polarity can regulate spindle positioning, which controls the site of contractile ring assembly and, consequently, the division plane (Grill et al., 2001; Maddox et al., 2007, 2012; Davies et al., 2014).

**Fig. 1.**
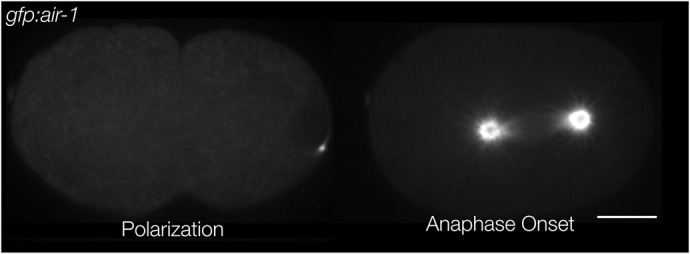
**Aurora A (AIR-1) accumulation during polarization and cytokinesis in *C. elegans* embryos**. AIR-1, which locally inhibits contractility, is enriched at the centrosomes. Anterior is positioned to the left. Scale bar: 10 µm.

**Fig. 2.**
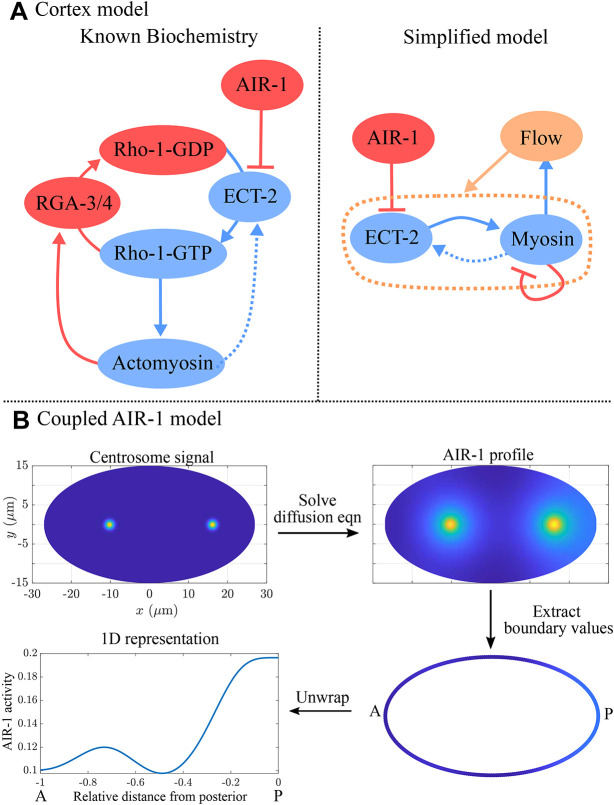
**Modeling schematic for this study.** (A) The model of the cortex, with the known biochemistry on the left and our simplified model on the right. This model takes the AIR-1 signal as input, and the equations are Eqn 1. (B) Procedure for determining the AIR-1 signal. Given centrosome positions (shown in cytokinesis in wild-type embryos), we solve for the AIR-1 profile on the cross-section using Eqn 2, then formulate a one-dimensional AIR-1 profile by extracting the values on the boundary.

The *Caenorhabditis elegans* zygote provides a powerful model in which to study both polarization and cytokinesis (Fig. 1). In *C. elegans*, as in many other animal cells, contractility in polarity establishment and cytokinesis is mediated by the GTPase RHO-1, which activates myosin through its effector Rho kinase. RHO-1 transitions between an active (GTP) state and inactive (GDP) state via interactions with the RhoGEF ECT-2 and RhoGAP RGA-3/4, which activate and inactivate RHO-1, respectively (Fig. 2A; Michaux et al., 2018; Basant and Glotzer, 2018). Contractility in polarization and cytokinesis relies on modulation of this circuit. During polarization, contractility is activated by a nematode specific protein, NOP-1, which appears to globally activate ECT-2 (Tse et al., 2012). Contractility is inhibited by centrosomes at the position of sperm entry, establishing the posterior pole and triggering anterior-directed cortical flows that facilitate the segregation of anterior and posterior PAR proteins into distinct domains (Goldstein and Hird, 1996; Munro et al., 2004; Lang and Munro, 2017; Gross et al., 2019). During cytokinesis, the centralspindlin complex accumulates at the mid-plane of the spindle, where it activates ECT-2 and RHO-1, thus promoting contractility (Glotzer, 2005; Basant and Glotzer, 2018). This pathway combines with a second pathway similar to polarization, i.e. activation of RHO-1 spatially modulated by the centrosomes (Tse et al., 2012). Given the positions of the separated centrosomes, this also biases contractility to the cell equator (White, 1985; Werner et al., 2007; Loria et al., 2012). The goal of this study was to use mathematical models to determine whether the known regulatory pathways are sufficient to explain these highly stereotyped behaviors.

Recent studies have characterized the mechanism by which polarizing and dividing cells pattern the RHO-1 activation pathway. During polarization, Aurora A kinase (AIR-1) associates with the sperm centrosome, which is the sperm-derived structure that promotes polarity establishment (Hannak et al., 2001; Cowan and Hyman, 2004; Klinkert et al., 2019; Kapoor and Kotak, 2019; Zhao et al., 2019; Longhini and Glotzer, 2022). Recent work (Longhini and Glotzer, 2022) showed that AIR-1 impacts cortical dynamics by inhibiting ECT-2. Specifically, ECT-2 dissociates from the posterior membrane in an AIR-1-dependent manner, and it contains consensus sites for AIR-1 that are required for AIR-1 responsiveness. During polarization, ECT-2 exhibits posterior depletion and anterior enrichment, a pattern of accumulation that requires cortical myosin flows. A similar set of events occur upon anaphase onset, coincident with cytokinesis (Longhini and Glotzer, 2022). Although the centrosomes have duplicated, matured, moved farther from the cortex (Fig. 1) and accumulated much more AIR-1 in cytokinesis, there remains a strong, ultra-sensitive dependence between the distance of the centrosome from the nearest cortical domain and the amount of cortical ECT-2 at that site; proximal centrosomes correlate with a reduction in cortical ECT-2 (Longhini and Glotzer, 2022).

Although the qualitative mechanisms by which AIR-1, ECT-2 and myosin interact to generate contractility and flow have thus been well characterized, how the hypothesized pathways could generate the quantitative patterns of myosin and ECT-2 accumulation during polarization and cytokinesis is still unclear. For instance, unlike the anterior PAR proteins, which have residence times on the order of 100 s (Robin et al., 2014), ECT-2 cannot be strongly advected, as it exchanges rapidly between the cytoplasm and the cortex on timescales of a few seconds, appearing to preferentially accumulate on the cortex at myosin-enriched sites (Longhini and Glotzer, 2022). Consequently, it remains unknown whether a short residence time, preferential recruitment by myosin and weak advection by cortical flows could combine to generate the observed asymmetric accumulation of ECT-2 during polarization. More generally, it is not known whether additional mechanisms are required to explain the pattern of ECT-2 accumulation during cytokinesis, as the centrosomes are so much further from the cortex at that stage.

In this study, we tested whether a minimal set of interactions can explain the dynamics of ECT-2 and myosin during both polarity establishment and centralspindlin-independent cytokinesis. To do this, we constructed a mathematical model that uses an AIR-1 signal, which diffuses from the centrosomes to the cortex, as an input to a continuum model of contractility (Fig. 2), which is similar to those previously described (Michaux et al., 2018; Gross et al., 2019). We show that our model can explain the initial dynamics of polarization, similar to those observed in the absence of PAR proteins. Furthermore, the same model reproduces the patterns of ECT-2 accumulation observed during cytokinesis, thus demonstrating a quantitative similarity between the two processes.

## RESULTS

We used the model to explore the quantitative similarities between polarization and cytokinesis. As discussed in the Materials and Methods section, some of the parameters are constrained using measurements from the polarized wild-type embryo. As such, the statements we make about wild-type polarization are shaped by existing data. Changing parameters, such as the centrosome–cortex distance and ECT-2 residence time, will demonstrate the importance of the centrosome position and relevance of rapid ECT-2 exchange during polarization. Perhaps more important is the model's direct extrapolation to cytokinesis, which concludes this section. Without changing any parameters, we demonstrate that the model can also explain the observed ECT-2 accumulation patterns during cytokinesis (Longhini and Glotzer, 2022), which are cued by larger centrosomes with 30-fold more AIR-1 than in polarization (Fig. 1).

### The centrosome distance determines the strength of polarization

**Fig. 3.**
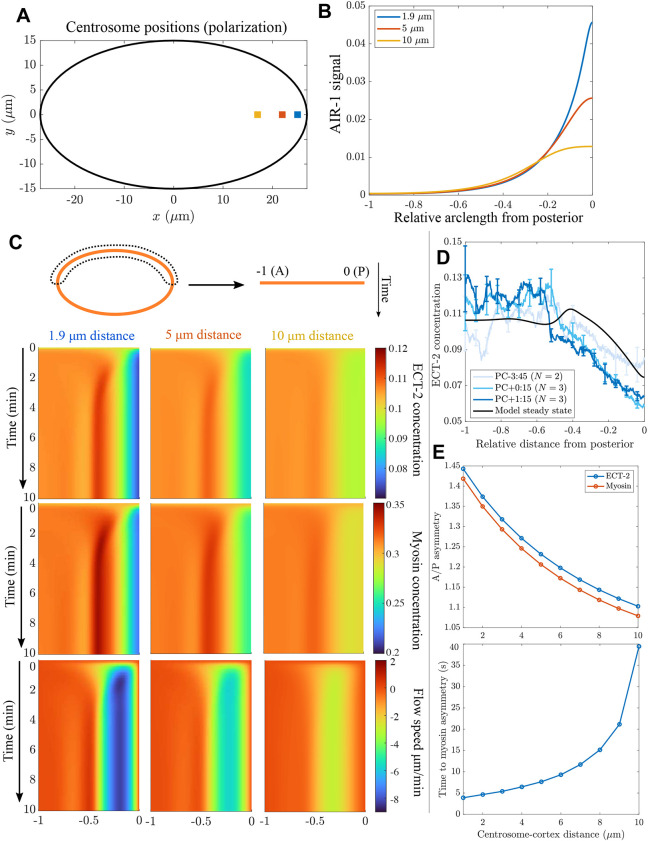
**Centrosome locations set polarization dynamics.** (A) The location of the centrosomes in our polarization simulations; we position both centrosomes 1.9, 5 and 10 µm away from the cell boundary. (B) The resulting AIR-1 signals along the cell perimeter. (C) The dynamics of polarization, starting from the uniform state, with the computed AIR-1 signals. We show the ECT-2 concentration (top), myosin concentration (middle) and flow speed (bottom) in pseudo-kymographs, with time on the *y-*axis and the anteroposterior (A/P) axis on the *x-*axis [anterior (A) at left and posterior (P) at right]. (D) The steady*-*state ECT-2 concentration in the model with centrosome–cortex distance of 1.9 µm, compared to experimental ECT-2 profiles in polarizing embryos. See Materials and Methods for data extraction procedure; the profiles are averaged over 2:30 (min:s) time intervals, with times in the legend being the middle of the interval (PC, pseudo-cleavage furrow formation). (E) The A/P asymmetry in myosin and ECT-2 after 10 min (top) and the time for symmetry breaking (bottom), both as a function of the centrosome–cortex distance.

During cell polarization, the newly duplicated centrosomes typically sit very close to the posterior cortex (∼1.9 µm away) and are small (approximate radius of 0.4 µm), as they have not yet accumulated large amounts of pericentrosomal material (Bienkowska and Cowan, 2012; Decker et al., 2011). To explore the effects of centrosome distance and location on AIR-1, we positioned centrosomes at a distance 1.9, 5 and 10 µm from the posterior pole, and measured the resulting AIR-1 signal by solving the diffusion equation (Eqn 2) on the embryo cross-section and extracting boundary values (see schematics in Fig. 2B and Fig. 3A). As expected, there is a significant decrease in the AIR-1 signal as the distance between the centrosomes and the cell boundary increases. Compared to a 1.9 µm distance, centrosomes 5 µm from the cortex exhibit a decrease of 50% in AIR-1, and those 10 µm from the cortex exhibit a further decrease of 50% (Fig. 3B).

Using these profiles of AIR-1 activity, we ran the cortex model forward in time to reach a steady state for polarization. For reference, in the absence of PAR proteins, the centrosomal signal induces a transient clearing of myosin from the posterior pole, and the myosin profile reverts back to a uniform state after the centrosomes move towards the cell equator (Gross et al., 2019; fig. 2E). Fig. 3C shows that our simulations reproduce the initial smaller-scale clearing of both myosin and ECT-2. Notably, the steady state ECT-2 accumulation in our simulation (which is steady because the centrosome positions are fixed) matches experimental data from early establishment phase (4 min before pseudo-cleavage), but diverges from the quasi-steady state that emerges once polarity is established (Fig. 3D), perhaps reflecting the influence of PAR proteins in the network (Gross et al., 2019). The predicted domain of ECT-2 clearance comprises ∼30% of the half-perimeter (∼20 µm on either side of the pole), which is in good agreement with experimental observations in PAR mutants (Gross et al., 2019; fig. 2E), suggesting that the model can effectively capture the transient posterior clearing induced by the AIR-1 signal when PAR proteins are absent.

In the model, changing the distance between the centrosomes and the cortex affects the quantitative values of the asymmetries and the time to reach them (Fig. 3E), but not the myosin peak location. As shown in the kymographs in Fig. 3C, the peak location, which correlates with the position of the pseudo-cleavage furrow (Reymann et al., 2016), is roughly the same across all conditions, because it is controlled by the hydrodynamic length scale (20% of the half-perimeter). Similar to previous observations (Bienkowska and Cowan, 2012; fig. 3F), the time for ‘symmetry breaking’, defined as a local (5%) clearance in myosin from the posterior pole, is tenfold higher when centrosomes are 10 µm away from the cortex than when they are 1 µm away. However, our model predicts an exponential scaling of the time to symmetry breaking at very large distances; this does not match the linear trend up to 10 µm that was previously reported (Bienkowska and Cowan, 2012; fig. 3F).

### Rapid exchange and indirect recruitment

**Fig. 4.**
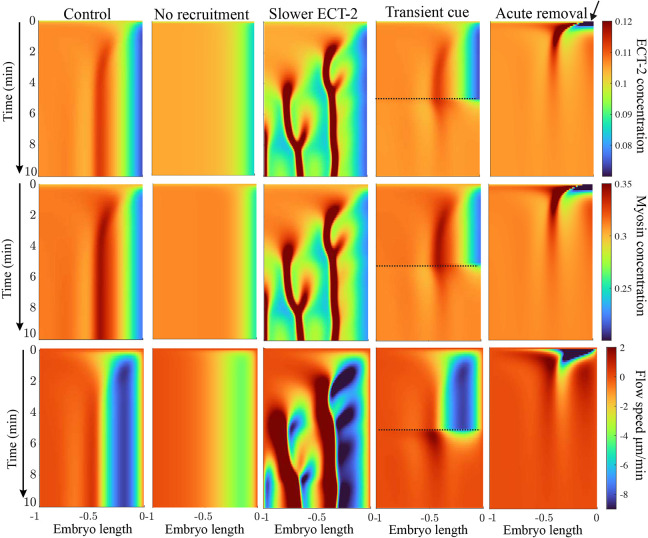
**Adjusting conditions in polarization.** First column: control parameters. Second column: simulating the same parameter set without myosin-mediated recruitment of ECT-2 (*k*_ME_=0). Third column: adjusting parameters so that, rather than be recruited by myosin, ECT-2 has a fourfold longer residence time and is only advected. Fourth column: simulating the case where the AIR-1 cue is present for 5 min (up to the dotted lines), after which we remove the AIR-1 signal and follow relaxation. Fifth column: simulating the case of no AIR-1 signal, but an initial condition in which ECT-2 and myosin concentrations are set to zero in a posterior region equal to 10% of the embryo (arrow).

We have shown that long-range redistribution of ECT-2 is possible despite its short residence time. The driver of this redistribution is our assumption of indirect recruitment of ECT-2 by a longer-lived species, which is advected by cortical flows. To explore how this assumption influences polarization kinetics, in Fig. 4 (first two columns) we considered two alternative models for how ECT-2 might segregate during polarization. In the first model, we remove recruitment of ECT-2 (by setting *k*_ME_=0 in Eqn 1a) and observe, at most, 10% clearing at the posterior. Indeed, with realistic flow speeds of at least 5 µm/min (Gross et al., 2019), molecules with residence time of 5 s can move at most 0.4 µm, which is less than 0.5% of the embryo perimeter, indicating that cortical flows alone are insufficient to reproduce typical ECT-2 clearance levels.

An alternative model, albeit inconsistent with measured rates of ECT-2 exchange, is one in which ECT-2 stably associates with longer-lived components on the cortex [for example through oligomerization (Illukkumbura et al., 2023)], so that the lifetime of ECT-2 is four times longer on the cortex. In this case, we observe contractile instabilities not seen in wild-type embryos. As in control conditions, a local maximum in ECT-2 initially forms near ∼30% embryo length. Because of the increased residence time, however, ECT-2 is also advected from the anterior into the peak, which creates a local minimum in myosin and drives the formation of a second, posterior peak. Thus, the rapid exchange of ECT-2 is important for stabilizing unidirectional flows during polarization. This outcome represents an experimentally testable prediction, whereby a version of ECT-2 with more stable binding kinetics should produce contractile instabilities.

### Assessing alternative polarity cues

The contractility cue that drives polarization establishment has been suggested to result from dynein-dependent removal of myosin (Chapa-Y-Lazo et al., 2020). Although experiments have shown that polarity establishment is dynein independent (Longhini and Glotzer, 2022), we nevertheless considered whether a transient reduction in contractility would be sufficient to trigger polarity establishment. In particular, we performed two simulations: one with the AIR-1 cue active for 5 min, and a second that lacks an AIR-1 cue but in which we acutely remove myosin and ECT-2 at the posterior pole. The resulting dynamics over 10 min are shown in Fig. 4. In both cases, transient cues or initial conditions ultimately relax to a steady state in which ECT-2 and myosin are nearly uniform and flow speeds are near zero.

The main difference between the transient AIR-1 cue and acute removal of myosin is in the intermediate dynamics. For a transient cue, the flow starts at zero and steadily increases over the first minute, peaking with a flow speed of 8 µm/min. Turning off the cue causes the flow speeds to slow below 2 µm/min in less than 1 min. By contrast, unloading myosin and ECT-2 from the posterior at *t*=0 triggers a similar set of flows towards the anterior, but the flows are maximal at *t*=1 min and then steadily decrease over time. Experimental data in PAR mutants show flows that reach a maximum velocity almost immediately after polarity triggering, but the magnitude (5 µm/min) of these flows persists throughout polarity establishment phase (3–4 min) (Gross et al., 2019; fig. 2G). Thus, this supports models in which AIR-1 triggers a local and persistent inhibition of contractility, similar to that reported previously (Gross et al., 2019).

The rapid recovery of ECT-2 and myosin in our transient cue simulation comes from the complete removal of the AIR-1 cue at *t*=5 min. Of course, the cue's removal is more gradual *in vivo* and corresponds to steady motion of the centrosomes away from the posterior cortex. In Fig. S8, we demonstrate that a much longer timescale of posterior recovery results when we model this (more realistic) case. The modeled recovery of myosin with relocalizing centrosomes compares well with previous results that measured myosin recovery in PAR-depleted embryos (Gross et al., 2019; fig. 2E).

### ECT-2 accumulation in cytokinesis

The asymmetric accumulation of ECT-2 on the cortex during cytokinesis is sensitive to the position of the two centrosomes (Longhini and Glotzer, 2022). A plot of ECT-2 accumulation as a function of distance from the anterior or posterior pole closest to the centrosome reveals an ‘S-shaped’ curve; at short and long distances there is a plateau in the ECT-2 accumulation (Longhini and Glotzer, 2022; fig. 7A). By contrast, for distances in the range 10–20 µm, there is an ultra-sensitive dependence of the ECT-2 concentration on the proximity. Note that during cytokinesis, there are two mature centrosomes that contain significantly more AIR-1 (∼30 times; Fig. S2) than the immature centrosomes that trigger polarity establishment.

**Fig. 5.**
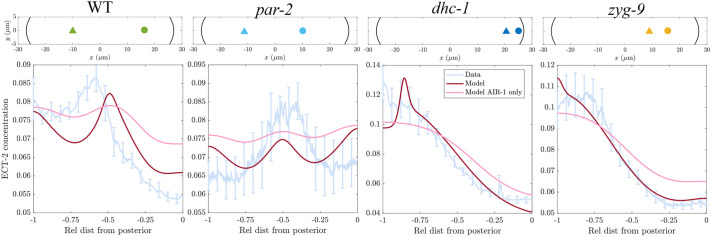
**Extending the model to infer ECT-2 profiles in cytokinesis.** We consider four experimental conditions from Longhini and Glotzer (2022): wild type (WT; *N*=10), *par-2*(RNAi) (*N*=5), *dhc-1*(RNAi) (*N*=10) and *zyg-9*(b244) (*N*=9). For each condition, we show the average centrosome positions in the top panel. The corresponding bottom panel shows the experimental ECT-2 profile, averaged over a 50 s window beginning at cleavage furrow formation, compared with the steady-state model result (dark red lines), and the model's steady state when ECT-2 is only impacted by AIR-1 and not myosin (*k*_ME_=0 and *v*=0 in Eqn 1a).

To test whether the model that accurately predicts polarization recapitulates the behavior of ECT-2 during cytokinesis, we modeled the behavior of ECT-2 and myosin using the centrosome positions measured previously (Longhini and Glotzer, 2022) and repeated in Fig. 5. We considered the case of wild-type embryos and three representative experimental conditions, two with asymmetric centrosome positions [*dhc-1*(RNAi) and *zyg-9*(b244)], and one [*par-2*(RNAi)] with symmetric centrosome positions. As in polarization, we solved the diffusion equation (Eqn 2) on the embryo cross-section with the given centrosome positions, then extract the AIR-1 signal (in arbitrary units) along the cell perimeter (Fig. S2). Despite a much (fourfold) stronger posterior AIR-1 signal in *dhc-1*(RNAi) embryos, the anteroposterior (A/P) asymmetry in ECT-2 accumulation only changes by ∼25% compared to *zyg-1*(b244); we used this observation to constrain the AIR-1 saturation level *A*_sat_ that appears in Eqn 1a (see Materials and Methods).

We used the computed AIR-1 signals as inputs to the same cortex model (Fig. 2A) that we parameterized under polarization conditions. Without changing any of the parameters, we simulated each experimental condition to steady state, then compared the profile of ECT-2 accumulation with the experimental data taken over a 50 s window beginning at cleavage furrow formation (multiplied by a constant to match the mean model concentration). For the four centrosome positions simulated, the model quantitatively reproduces the ECT-2 accumulation pattern (Fig. 5; see Fig. S9 for AIR-1 depletion with wild-type centrosome positions). In wild-type and *par-2*(RNAi) embryos, the model qualitatively reproduces the observed profile but tends to underestimate the A/P asymmetry. The shift in the central peak is likely due to centralspindlin-directed ECT-2 accumulation (Longhini and Glotzer, 2022), which is not accounted for in the model. In all experimental conditions, the fit to the data significantly degrades when we remove cortical flows and indirect recruitment (setting *v*=0 and *k*_ME_=0 in Eqn 1a). Thus, as during polarization, cortical flows amplify weaker AIR-1 signals. Indeed, as shown in Fig. S9, the predicted ECT-2 profile without myosin flows and indirect recruitment matches experimental data with partial myosin depletion, indicating that the model correctly accounts for both the response to AIR-1 signals and flow-based amplification (albeit imperfectly in some cases).

## DISCUSSION

Cell polarization in the *C. elegans* zygote is dependent on a centrosome-dependent signal that locally inhibits contractility. Similarly, cytokinesis can be influenced by aster positioning (White, 1985; Dechant and Glotzer, 2003; Severson and Bowerman, 2003; Munro et al., 2004). Only recently, however, has a better understanding of the nature of these cues emerged (Gross et al., 2019; Kapoor and Kotak, 2019; Klinkert et al., 2019; Zhao et al., 2019; Longhini and Glotzer, 2022; Manzi et al., 2024). In particular, a recent study (Longhini and Glotzer, 2022) showed that AIR-1, which accumulates and is presumably activated at centrosomes, causes inhibition of the RhoGEF ECT-2 (and, consequently, RHO-1 and myosin) at the proximal cortex. The goal of this study was to determine whether a minimal mathematical model could explain experimental observations in both polarization and cytokinesis.

We introduced a hybrid two- and one-dimensional model (Fig. 2), in which the centrosome positions are obtained from experimental data, and used it to obtain a cross-sectional profile of AIR-1 activation. Assuming diffusion of AIR-1 to the cortex (boundary of the cross-section), we then obtained the profile of AIR-1 as an input to a model of cortical contractility. This cortex model, which we defined on the cross sectional-boundary and consequently made one dimensional, included negative feedback of AIR-1 on ECT-2 accumulation, and positive feedback of myosin on ECT-2 accumulation, both through advection and recruitment by advected species. The coupling of the AIR-1 and cortex models allowed the dynamics of the active cortex to be dictated by the positions of the centrosome(s). We constrained the model using experimental observations (Longhini and Glotzer, 2022) and assuming modest (at most, twofold) effects on cortical ECT-2 by AIR-1 (negatively) and myosin (positively). The parameters arising from these constraints placed the model in a regime that can amplify small signals without yielding to contractile instabilities (Fig. S4).

This minimal model recapitulated many of the pertinent observations from polarization and cytokinesis. It produced transient polarization (in the absence of PAR proteins) with the observed flow speeds and protein ratios, and revealed the need for a persistent AIR-1 cue over time to maintain a polarized state in the absence of PAR reorganization. The picture that emerged from our experiments and modeling is a dynamic ECT-2 molecule that rapidly exchanges with the cortex, being preferentially recruited by longer-lived, flow-coupled molecules to sustain polarization. In this way, it reproduces other examples from cell biology in which stable configurations are mediated by transient interactions (Ladurner et al., 2016). The rapid exchange of ECT-2 and, to a lesser extent, myosin gives the model a quasi-steady nature; changes in the distribution of AIR-1 rapidly establish a new steady state. These dynamics are consistent with previous experimental results (Longhini and Glotzer, 2022; fig. 2A), which showed an acute response of cortical ECT-2 to spindle rocking during anaphase on a timescale of 10 s and could help to explain the rapid repositioning of the cleavage furrow in response to spindle displacements (Rappaport, 1985).

It is instructive to contrast the role of ECT-2 in generating cortical flows to that of the anterior PAR proteins, specifically PAR-3. Although it has long been known that reduced PAR-3 levels correlate with reductions in cortical flows, these changes have only recently been linked to the residence time of PAR-3 molecules on the membrane (Chang and Dickinson, 2022; Illukkumbura et al., 2023). In wild-type embryos, PAR-3 monomers (which have residence time less than 1 s) (Lang et al., 2024 preprint) oligomerize to stably bind the membrane (residence times on the order 100 s), which allows them to both create and be advected by cortical flows (Munro et al., 2004). Consequently, embryos with oligomerization-defective PAR-3 fail to polarize because of a lack of coupling to (weaker) cortical flows. Our analysis indicates that ECT-2 lives in a different part of the stability diagram of chemical–mechanical coupling. In wild-type embryos, the ECT-2 exchange kinetics [as measured by fluorescence recovery after photobleaching (FRAP)] are on the order of a few seconds, yet large-scale cortical flows are generated. Consequently, the model predicted that a longer ECT-2 residence time would produce hypercontractility, specifically a counterflow from the anterior end of the cell that prevents proper polarization. This result represents an important prediction that can be tested experimentally. The differences in stability behavior might be due to the coupling of ECT-2 and PAR-3 to myosin; whereas the role of PAR-3 in generating flows is likely indirect, ECT-2 directly generates flows by activating RHO-1 and myosin. Thus, more tunable control of contractility could be achieved by faster turnover rates in the latter case.

The question that underpins our work is how a persistent flow could affect the distribution of transiently bound proteins, independent of the underlying biochemical circuit in which they operate. For PAR-3, the typical measured diffusivities (0.01 µm^2^/s) and residence times (200 s) are insufficient to explain the measured asymmetries when only advection and diffusion are assumed to contribute to patterning (Illukkumbura et al., 2023; fig. 6H). This problem only worsens with ECT-2, which is apparently patterned by myosin-mediated flows, despite having a residence time on the order of a few seconds (Longhini and Glotzer, 2022). Here, we found that introducing recruitment of ECT-2 by a longer-lived species (or any species that is advected by and colocalizes with myosin) could result in similar patterning as cortical flows. Given that we previously showed that ECT-2 only segregates when its PH domain is intact (Longhini and Glotzer, 2022; fig. 5A), we speculate that myosin could advect factors that cause PIP2 to concentrate anteriorly (Scholze et al., 2018; Hirani et al., 2019; Nakayama et al., 2009), which would then contribute to ECT-2 recruitment. This model could explain how ECT-2 segregates while turning over rapidly.

Because the model parameters were tuned to match observations during polarization, it was most striking that the model also predicted patterns of cortical ECT-2 accumulation in cytokinesis across multiple experimental conditions solely by modifying centrosome size and positions (Dechant and Glotzer, 2003; Verbrugghe and White, 2007; Longhini and Glotzer, 2022). Similar to recent work on contractility during cytokinesis (Werner et al., 2024), we found that the accumulation of ECT-2 in different conditions could be better reproduced by incorporating mechanochemical feedback to amplify the AIR-1/ECT-2 signal. Historically, inhibition of cortical contractility by asters was thought to be microtubule mediated, given their proximity to the cortex (Dechant and Glotzer, 2003; Motegi et al., 2006). Yet experimental evidence (Klinkert et al., 2019; Kapoor and Kotak, 2019; Zhao et al., 2019; Longhini and Glotzer, 2022) and this mathematical model indicate that centrosome position and embryo geometry (i.e. the ability for AIR-1 to diffuse from the centrosomes to the cortex) serve as the primary determinants of polar relaxation. However, as cortical interactions with astral microtubules frequently control spindle and hence centrosome positioning (Grill et al., 2001; Schaefer et al., 2000; Tame et al., 2014), microtubules nevertheless play a role in this process.

## MATERIALS AND METHODS

In *C. elegans* embryos, the AIR-1 signal originates from the centrosomes, which are positioned in the interior of the three-dimensional cell (Fig. 1), while the contractile dynamics occur on the two-dimensional cell cortex (boundary). For our model, we consider a cross-section of the embryo, so that the AIR-1 dynamics occur in two dimensions, and the contractile dynamics occur on the one-dimensional boundary (Fig. 2B). The workflow is to first set the centrosome positions according to experimental data (Fig. 1), then solve a diffusion equation to obtain the AIR-1 profile at the cortex. This becomes an input to a set of one-dimensional reaction–diffusion–advection equations that treat the ECT-2/myosin relationship. In order to study how the cortex responds to the expected AIR-1 signals in polarization and cytokinesis, we assume that the centrosomes (and AIR-1 signal) are fixed (see Fig. S8 for a simulation that relaxes this assumption).

### Basic model of contractility

At the cortex of the *C. elegans* zygote, AIR-1 inhibits accumulation of ECT-2 by increasing its dissociation rate through phosphorylation. The cortical pool of ECT-2 gains the ability to activate RHO-1, which activates myosin (Fig. 2A). Myosin feeds back on ECT-2 through advection by cortical flows, and there is nonlinear negative feedback of myosin accumulation (through RGA-3/4-dependent inactivation of RHO-1) (Michaux et al., 2018). To translate these dynamics into a simple model (Fig. 2B), we neglect the intermediary of RHO-1 and formulate a model with two variables: *E* (for ECT-2) and *M* (for myosin). In dimensional units, the equations we use are as follows:
(1rma)

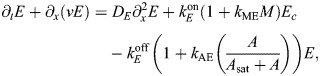

(1rmb)

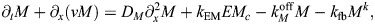

(1rmc)

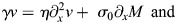

(1rmd)

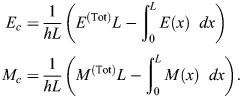
Similar to previous studies using these types of models (Goehring et al., 2011; Dawes and Munro, 2011; Kravtsova and Dawes, 2014; Gross et al., 2019), the model geometry is one dimensional and can be viewed as a one-dimensional slice of the cell cortex combined with a well-mixed cytoplasm (see Fig. 2B). Each species evolves by advection by cortical flows [terms *∂_x_*(*vE*) and *∂_x_*(*vM*)], diffusion in the cortex (terms *D_E_∂_x_*^2^*E* and *D_M_∂_x_*^2^*M*) and binding and unbinding from the cortex. The binding rate is also proportional to the cytoplasmic concentration of each protein, defined in Eqn 1d, where *L* is the domain length, *h* is the cytoplasmic ‘thickness’ (so that *hL* is the total area), and *E*^(Tot)^ is the concentration of ECT-2 when all of it is bound to the cortex (likewise for *M*) (Lang and Munro, 2022). Finally, the velocity equation (Eqn 1d) expresses the balance of active stress (which we assume is proportional to myosin concentration) with viscous stress and frictional resistance (Mayer et al., 2010). For simplicity, along with many other components, we do not model the actin network, a topic for future study.

The binding/unbinding terms in the ECT-2 and myosin equations rely on the following major assumptions:

1. The variable *E* represents unphosphorylated, active ECT-2 bound to the cortex. We do not consider phosphorylation of ECT-2 in the cytoplasm and instead assume that the effective ECT-2 binding rate (which we fit to experimental data) represents the binding rate of unphosphorylated ECT-2. Consistent with this assumption, the negative flux in ECT-2 represents the combined rate of unbinding and phosphorylation, with the latter being proportional to the AIR-1 concentration, except at high AIR-1 when it saturates (see section ‘Coupled model of AIR-1'). Likewise, we assume that activation of ECT-2 by NOP-1 occurs uniformly throughout the cortex (Tse et al., 2012).
2. It was previously shown that ECT-2 has a small residence time at the cortex (on the order of a few seconds) (Longhini and Glotzer, 2022). Under these conditions, we show in Fig. S4 that the direct transport of ECT-2 by flows contributes negligibly to its steady-state profile. It was previously speculated that ECT-2 could be effectively ‘transported’ by associating with other components that are more stably bound to the cortex (Longhini and Glotzer, 2022). We incorporate this assumption into our model by assuming recruitment of ECT-2 by a species that is advected by cortical flows. For simplicity, in our equations, we assume that the concentration of this species is equal to that of myosin, thus giving the *k*_ME_*ME_c_* term in the ECT-2 equation (Eqn 1a). In Fig. S7, we show that explicitly introducing a third species, which recruits ECT-2, into the equations gives similar patterns during polarization.
3. Based on previous work that demonstrated an important role for RhoGAP in setting the size of the anterior domain in polarizing embryos (Schonegg et al., 2007), combined with other work showing a nonlinear relationship between RhoGAP activity and Rho/myosin accumulation (Nishikawa et al., 2017; Michaux et al., 2018), we postulate nonlinear negative feedback in the myosin kinetics, with an inactivation rate proportional to *M^k^*. As long as *k*>1, this term provides a way of controlling potential instabilities that arise in the simple active gel model (Eqn 1c) (Nishikawa et al., 2017). In the main text, we present results using *k*=2, but in Fig. S5 we show that model predictions are similar when *k*=3 instead.

### General process of parameter estimation

As described in the ‘Details of model parameterization' section, we convert the model equations to dimensionless form, then fit the parameters using a combination of direct experimental measurements and inference based on other experimental data. The overall flow of the parameter fitting process goes as follows:

1. We first assign values to the diffusivities and unbinding rates of each component that come from direct experimental measurements (Gross et al., 2019; Goehring et al., 2011; Michaux et al., 2018).
2. Using previously imaged embryos with myosin and ECT-2 markers (Longhini and Glotzer, 2022; fig. 1), we measure the effective myosin and ECT-2 profiles during pseudo-cleavage (Fig. S3A–C), which we treat as a quasi-steady state.
3. Based on the quasi-steady myosin profile, we fit the velocity parameters in Eqn 1c to match bulk flow speeds in wild-type embryos (which are typically at most 10 µm/min) (Gross et al., 2019) (Fig. S3D).
4. To fit the parameters in the myosin equation (Eqn 1b), we impose the measured ECT-2 profile and adjust *k*_fb_ and *k*_EM_ until we match the experimentally measured myosin profile (Fig. S3E).
5. We use previous measurements in myosin-depleted embryos (Longhini and Glotzer, 2022; fig. 3A) to infer how AIR-1 impacts ECT-2 in the absence of myosin (i.e. to fit *k*_AE_) (see Fig. S4A).
6. With all other parameters fixed, we increase the rate (*k*_ME_) at which myosin (or a species associated with myosin) recruits ECT-2 until we reach the boundary of contractile instabilities. We choose a value for *k*_ME_ that sits near the boundary between the stable and unstable regime, without giving unstable behavior (Fig. S4). The result of this parameter fitting is that ∼40% of the ECT-2 that binds to the cortex is recruited by myosin-associated species.

Although it is tempting to equate the model's myosin-driven, contractile instabilities with pulsatile RHO-1/myosin excitability (Nishikawa et al., 2017), the latter are actually myosin independent (Michaux et al., 2018), which illustrates that the wild-type embryo does not sit in the model's fully unstable regime. In fact, the overall speed of bulk flows does not depend on pulsatility of RHO-1 (Michaux et al., 2018; fig. 7), affording a biological justification for modeling the simpler case in which these well-characterized pulses of RHO-1 activation are absent (or, more precisely, the case in which these randomly positioned pulses are averaged over many cross-sections to yield a steady signal). It was recently shown that knockdown of the downstream effectors of RHO-1 could cause contractile instabilities, which implies that the wild-type *C. elegans* embryo likely sits near, but not within, the unstable regime (Yao et al., 2022). In this way, a comparison can be drawn to the *Xenopus* model system, where normally quiescent oocytes can exhibit excitable dynamics through overexpression of ECT-2 and RGA-3/4 (Michaud et al., 2022; Chen et al., 2024).

### Coupled model of AIR-1

As an input to the cortex model, we solve for the AIR-1 profile on the boundary (the cortex) of a two-dimensional embryo cross-section by specifying the position of the centrosome(s) and solving a diffusion equation in the embryo interior. Letting *a*(*x*) be the concentration of AIR-1 in the two-dimensional embryo cross-section, its diffusion in the cytoplasm is described by
(2rma)

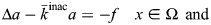

(2rmb)

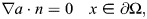
where Eqn 2a is the diffusion equation for the concentration and Eqn 2b is a no-flux boundary condition through the boundary (here, Ω represents the embryo area and *∂*Ω represents the boundary). The signal *f*(*x*) comes from the two centrosomes, which we model by Gaussian densities:
(2rmc)

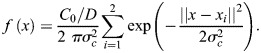
Here *x*_*i*_=(*x*_*i*_, 0) is the location of the *i*th centrosome, which changes depending on the experimental conditions. In addition to the centrosome location, the signal has two other parameters: *C*_0_/*D* is the strength of the cue [the integral of *f*(*x*) over the entire embryo cross-section, normalized by the cytoplasmic diffusion coefficient *D*], and 2*σ_c_* is roughly the centrosome ‘size’. For cytokinesis, the centrosomes have a radius of ∼1.4 µm, so we set *σ_c_*=0*.*7 µm. In polarization, the centrosomes have radius of ∼0.35 µm, so we set *σ_c_*=0*.*175 µm (Decker et al., 2011; fig. 1C). The absolute signal strength *C*_0_*/D* is arbitrary, but ratios between cytokinesis and polarization are well defined. Consequently, we set *C*_0_*/D*=1 for cytokinesis and *C*_0_*/D*=1*/*32 for polarization, according to our experimental measurements (Fig. S2).

The diffusion equation Eqn 2a also contains a basal rate of inactivation of AIR-1 (phosphatase activity). This introduces another parameter – the inactivation rate relative to the diffusion coefficient in the cytoplasm (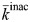, units µm^−2^). As shown in Fig. S1, low levels of phosphatase activity give high global AIR-1 levels, which translate to low ECT-2 levels everywhere. Such levels were shown to block pseudo-cleavage in centralspindlin-independent cytokinesis, owing to low contractility (Afshar et al., 2010; Kotak et al., 2016). We choose the phosphatase activity level 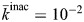 such that centrosomes close to the posterior pole (in polarization) negligibly impact the anterior domain (<1% of the posterior concentration) (see Fig. S1).

To infer the profile of AIR-1 during cytokinesis for the four experimental conditions shown in Fig. 5, we use the corresponding centrosome positions, set 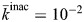, and solve Eqn 2. The resulting AIR-1 profile along the embryo perimeter is shown in Fig. S2. Despite large (fourfold) differences in posterior *zyg-9* and *dhc-1* AIR-1 signal, the A/P asymmetry between the two conditions increases by only 25% (Fig. 5). Because of this, we conclude that the saturation level of AIR-1 activity, *A*_sat_ in Eqn 1a, must lie near the *zyg-9* posterior levels. As such, we set *A*_sat_=0*.*25.

We use a standard first-order finite element method to solve Eqn 2a. In brief, the elliptical domain of the embryo is meshed into nodes and triangles (Persson and Strang, 2004), and the finite element matrix equation becomes 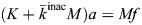, where *M* is the mass matrix and *K* is the stiffness matrix for finite elements, which are assembled using standard techniques (Gockenbach, 2006). Solving for *a*(*x*) everywhere gives a profile on the embryo perimeter (cortex), which we substitute into Eqn 1 as the one-dimensional profile *A*(*x*), using linear interpolation to map from the (irregular) finite element boundary nodes to a regular grid. Once it reaches the cortex, AIR-1 inhibits ECT-2 by increasing its cortical dissociation rate, in correspondence with experimental data in myosin-depleted embryos (Longhini and Glotzer, 2022; fig. 3A).

### Details of model parameterizaton for the coupled system

Because absolute concentrations are unknown, it is easiest to assign values to unknown parameters when they are in dimensionless form. To do this, we non-dimensionalize (Eqn 1) so that length is in units of the embryo perimeter *L*, time is in units of the bound myosin lifetime 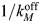, velocity is in units of 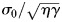 (Bois et al., 2011), and concentration of species *A* is in units of *A*^(Tot)^. This gives new dimensionless variables (denoted by carets),

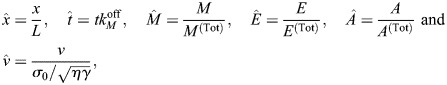
and a corresponding set of equations:
(3rma)

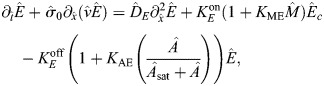

(3rmb)

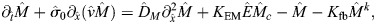

(3rmc)

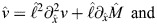

(3rmd)

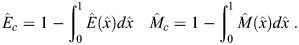
The conversion from dimensional to dimensionless form is given for all parameters in Table S1. Most of these conversions are straightforward, but there are some important parameters to highlight. In flow patterns, 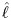 is a hydrodynamic length scale (scaled by domain perimeter) expressing the connectivity of the cortex; local disturbances in myosin will typically propagate at most a distance 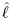. The parameter 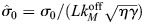 expresses the strength of the flows; the dimensional velocity in µm/s can be extracted by taking 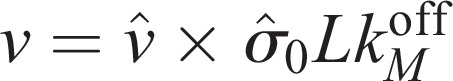.

We use standard numerical methods to solve Eqn 3. We discretize the one-dimensional domain at *N* points with spacing 1*/*Δ*x*, and define the centered differentiation matrix *D* and standard three-point Laplacian differentiation matrix Δ. Given the myosin profile at time step *n*, we first compute the velocity *v*^(*n*)^ by solving 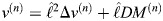. Once the velocity is computed, the ECT-2 and myosin equations are solved by combining a first-order upwind finite volume scheme for the advection terms (Hundsdorfer et al., 2003; sec. 1.4) with implicit treatment of the diffusion terms (using the standard three-point Laplacian). The reaction terms are all treated explicitly, and time stepping is first-order accurate.

Parameter estimation for the dimensionless equations (Eqn 3) can be performed in three steps: first, we directly assign quantities that have already been measured experimentally. Second, we freeze the ECT-2 profile and assign parameters to the myosin equation (Eqn 3b) to match experimental data. Third, we choose the parameters in the ECT-2 equation (Eqn 3a) based on stability considerations.

#### Direct measurements

Some of the parameters can be determined directly from experimental measurements, as follows:

1. The embryo cross-section is an ellipse with approximate radii 27 µm and 15 µm, which gives a perimeter *L*=134 µm (Goehring et al., 2011).
2. The variable 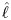 is the hydrodynamic length scale. In dimensional units, this was measured to be *ℓ*≈13 µm (Mayer et al., 2010), which means that 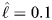 in Eqn 3c (10% domain perimeter).
3. The myosin-bound lifetime is ∼15 s, according to measurements in the anterior of wild-type embryos, or in Par mutant embryos, which do not polarize (Gross et al., 2019; fig. 1B). Because we typically model polarity establishment, where embryos are initially unpolarized, we set 
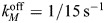
.
4. We assume that all species have a dimensional diffusion coefficient *D_E/M_*=0*.*1 µm^2^/s (Goehring et al., 2011; Gross et al., 2019; Robin et al., 2014). Rescaling length by *L* and time by 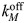 gives a dimensionless coefficient 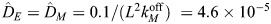. This dimensionless coefficient is sufficiently small as to render diffusion relatively unimportant in shaping the concentration fields. If we assume instead, for instance, that myosin cannot diffuse in the membrane, while ECT-2 has a tenfold larger diffusion coefficient, the steady-state profiles of ECT-2 and myosin are changed by, at most, 5% (see Fig. S6).
5. The ECT-2 lifetime was measured using FRAP to be on the order of a few seconds (Longhini and Glotzer, 2022; fig. 3D). In cytokinesis, we set 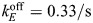, for a 3 s lifetime. Rescaling gives 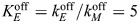. The data show slightly faster recovery during polarization, so we increase 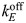 by 20% for those simulations.

#### Parameters for myosin equation

To infer the myosin parameters 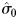, *K*_EM_, *k* and *K*_fb_, our strategy is to impose the ECT-2 profile measured experimentally at the quasi-steady state of pseudo-cleavage, then solve for the myosin parameters required to match the experimental myosin profile. As shown in Fig. S3, we utilize previously imaged embryos with ECT-2 and myosin reporters (Longhini and Glotzer, 2022; fig. 1) for this purpose. For each set of images, we use MATLAB's built-in algorithms to segment the embryo boundary and compute an arclength parameterization of the boundary curve. Following this, we use Fourier filtering to filter the result and obtain a smoothed boundary with a normal vector at each point (see Fig. S3A,B). For each point on the arclength curve, we draw a 30 pixel (3 µm) line inward and compute the maximum intensity along this line. Averaging over all frames in which pseudo-cleavage is present, then repeating for three embryos to generate error bars, gives the curves shown in Fig. S3C. Fitting these curves with a Fourier interpolant then gives smoothed representations that can be used for fitting. We note that the punctate myosin profile during establishment phase can somewhat confound the myosin measurements, but our data show a clear general trend that is captured by the Fourier interpolant.

To transition the target curves to model inputs, we scale by the expected amount of bound protein. Experimental data (Longhini and Glotzer, 2022; fig. 1) show that ∼10% of ECT-2 is bound to the cortex. This estimate is based on Fig. S3C, which shows the average ECT-2 intensity in the cortical region to be ∼1.5 times that in the cytoplasm. If the embryo is an ellipsoid with radii 27×15×15 µm, and the cortex has thickness 400 nm, then the cortex is 6.7% of the total volume. Multiplying by 1.5 gives 10% of the total ECT-2 bound. Although the specific number (10%) is of little consequence to our model, the relative abundance of ECT-2 in the cytoplasm demonstrates that cytoplasmic depletion will not play a role in the dynamics. A similar set of data (Gross et al., 2019; Figs S2 and S3) show that ∼30% of myosin is bound to the cortex. With these parameters, we scale the smoothed curves to obtain target curves for the model (the imposed ECT-2 curve is the experimentally measured curve, but scaled to have mean 0.1).

To infer the velocity strength 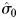 (Fig. S3D), we fix the myosin profile, then solve Eqn 3c to get the velocity profile and convert to µm/min. Previous work (Gross et al., 2019; fig. 3J) found the velocity in wild-type embryos to be at most 10 µm/min. For this reason, we set 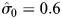 (Fig. S3D). Following this, we use the observed ECT-2 profile to set the remaining myosin parameters. We fix 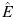 at the measured experimental profile, then solve the myosin equation (Eqn 3b) with three different values of *k*=1, 2, 3 to fit *K*_EM_ and *K*_fb_ to match the target myosin profile. When *k*=1, the solution to Eqn 3 can be written (neglecting diffusion and advection) as follows:

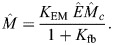
Because advection will only accentuate asymmetries, the minimum myosin asymmetry (maximum/min) that results from the imposed ECT-2 profile is 1.9 (equal to the ECT-2 asymmetry). Because the asymmetry in the myosin data (Fig. S3C) is 1.5, we need nonlinear inhibition (*k>*1 in Eqn 4e) to match the myosin profile. As shown in Fig. S3E, using *K*_EM_=9 and *K*_fb_=3*.*6 for *k*=2, while *K*_EM_=6*.*5 and *K*_fb_=5*.*25 for *k*=3 allows the solution of Eqn 3 to match the smoothed experimental data.

#### Fitting the ECT-2/AIR-1 parameters

There are now four parameters remaining: *K*_AE_, *A*_sat_, 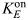 and *K*_ME_. The saturating AIR-1 threshold *A*_sat_ cannot be fit from polarization conditions because AIR-1 signals are low; as such, we set *A*_sat_=0*.*25 according to cytokinesis measurements (see Materials and Methods). The parameter *K*_AE_ can be set by simulating AIR-1 activity in the absence of myosin. In myosin-depleted embryos, it was previously shown that the ECT-2 asymmetry during early polarization is 1.2 (Longhini and Glotzer, 2022; fig. 3A), which implies that the local AIR-1 activity induces a 20% depletion of AIR-1 on the posterior. We therefore infer *K*_AE_ by simulating polarization (as in Fig. 3) with no myosin activity (setting *K*_ME_=0 and 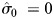). As shown in Fig. S4A (black lines), the steady state ECT-2 asymmetry is 1.2 when *K*_AE_=1*.*3.

With the AIR-1 parameters set, there is effectively one parameter remaining: the strength of indirect recruitment of myosin on ECT-2 (there is also *K_E_*^on^, the basal ECT-2 binding rate, but this is set to maintain 10% bound ECT-2 on the cortex). We take a systematic approach to setting this parameter: as shown in Fig. S4, setting *K*_ME_=0, so that the only interaction of ECT-2 with myosin comes via flows, gives a negligible change in the ECT-2 profile from the AIR-1-only case (no myosin). Thus, indirect recruitment must be responsible for shaping the ECT-2 profile. To fit a value, we increase *K*_ME_ until we trigger locally oscillatory patterns of ECT-2 accumulation. As shown in Fig. S4, these patterns occur upon the transition to the regime where the uniform ECT-2 profile is linearly unstable (see the next section for an analysis). The values we use are *K*_ME_=2*.*5 (for *k*=2) or *K*_ME_=1*.*5 (for *k*=3).

#### The parameters are on the edge of the stability boundary

To perform linear stability analysis of the model equations (Eqn 3a–d) with *K*_AE_=0, we perturb the myosin and ECT-2 profiles around the uniform state by setting 
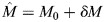
 and 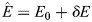, where 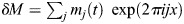, and likewise for *δE*. Substituting this representation for *M* into the velocity equation (Eqn 3c) then gives a representation for the velocity 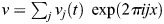, where

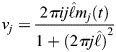
(Bois et al., 2011; eqn 11). Substituting this representation into the ECT-2 and myosin equations, and keeping terms to linear order in *δ* gives the following matrix equation:

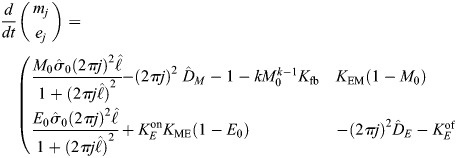
The dynamics are unstable if the 2×2 matrix above has a positive eigenvalue (negative determinant). The stability diagram in Fig. S4 shows the number of unstable modes for each pair (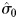, *K*_ME_), with all other parameters fixed to their default values (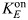 is adjusted to maintain 10% ECT-2 bound). The most unstable behavior (quantified by how many of the first ten Fourier modes *j*=1, … 10 are unstable) occurs for high flow speeds and high recruitment rates.

### Extended model with explicit intermediary

To more thoroughly examine our hypothesis that ‘myosin’ recruits ECT-2, we consider an explicit model where a third species (‘*P*’) is advected with myosin and recruits ECT-2. The dimensional equations governing this situation are as follows:
(4rma)

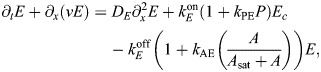

(4rmb)

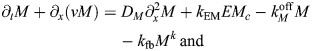

(4rmc)

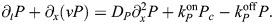
Similar to Eqn 3, we non-dimensionalize these equations to obtain the following system:
(4rmd)

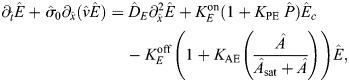

(4rme)

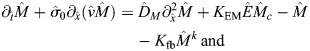

(4rmf)

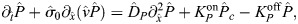
where 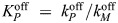, the binding rate 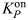 is arbitrary (we set it such that 30% of protein is bound, similar to myosin), and all parameters are the same as previously. In Fig. S7, we plot the corresponding steady ECT-2 profiles that result during polarization under different values of *D_P_*, 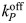
and *K*_PE_. To advect *P* with cortical flows, we consider residence times similar to those of the longest-lived PAR proteins, 
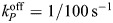
 and 
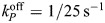
 (Robin et al., 2014; Illukkumbura et al., 2023). Similar to the more minimal model (Fig. S4), a small amount of recruitment propagates AIR-1- and flow-driven asymmetries, but larger coefficients lead to instabilities and oscillatory profiles. More specifically, we find that this model of recruitment through an advected intermediary typically undergoes instabilities at smaller levels of posterior clearance than the minimal model (Eqns 1, 3) which utilizes recruitment by species that colocalize with myosin. Nevertheless, the results for high diffusivities and intermediate residence times of the intermediary (bottom-right plot in Fig. S7) are quite similar to the model we consider in the main text.

## Supplementary Material



10.1242/joces.264093_sup1 Supplementary information

## References

[JCS264093C1] Afshar, K., Werner, M. E., Tse, Y. C., Glotzer, M. and Gönczy, P. (2010). Regulation of cortical contractility and spindle positioning by the protein phosphatase 6 PPH-6 in one-cell stage *C. elegans* embryos. *Development* 137, 237-247. 10.1242/dev.042754 20040490 PMC2799158

[JCS264093C2] Basant, A. and Glotzer, M. (2018). Spatiotemporal regulation of RhoA during cytokinesis. *Curr. Biol.* 28, R570-R580. 10.1016/j.cub.2018.03.045 29738735 PMC6508076

[JCS264093C3] Bienkowska, D. and Cowan, C. R. (2012). Centrosomes can initiate a polarity axis from any position within one-cell *C. elegans* embryos. *Curr. Biol.* 22, 583-589. 10.1016/j.cub.2012.01.064 22425158

[JCS264093C4] Bois, J. S., Jülicher, F. and Grill, S. W. (2011). Pattern formation in active fluids. *Biophys. J.* 100, 445a. 10.1016/j.bpj.2010.12.2620 21405254

[JCS264093C5] Chang, Y. and Dickinson, D. J. (2022). A particle size threshold governs diffusion and segregation of PAR-3 during cell polarization. *Cell Rep.* 39, 110652. 10.1016/j.celrep.2022.110652 35417695 PMC9093022

[JCS264093C6] Chapa-Y-Lazo, B., Hamanaka, M., Wray, A., Balasubramanian, M. K. and Mishima, M. (2020). Polar relaxation by dynein-mediated removal of cortical myosin II. *J. Cell Biol.* 219, e201903080. 10.1083/jcb.201903080 32497213 PMC7401816

[JCS264093C7] Chen, S., Seara, D. S., Michaud, A., Kim, S., Bement, W. M. and Murrell, M. P. (2024). Energy partitioning in the cell cortex. *Nat. Phys.* 20, 1824-1832. 10.1038/s41567-024-02626-6 PMC12889973 41675559

[JCS264093C8] Cowan, C. R. and Hyman, A. A. (2004). Centrosomes direct cell polarity independently of microtubule assembly in *C. elegans* embryos. *Nature* 431, 92-96. 10.1038/nature02825 15343338

[JCS264093C9] Davies, T., Jordan, S. N., Chand, V., Sees, J. A., Laband, K., Carvalho, A. X., Shirasu-Hiza, M., Kovar, D. R., Dumont, J. and Canman, J. C. (2014). High-resolution temporal analysis reveals a functional timeline for the molecular regulation of cytokinesis. *Dev. Cell* 30, 209-223. 10.1016/j.devcel.2014.05.009 25073157 PMC4245203

[JCS264093C10] Dawes, A. T. and Munro, E. M. (2011). PAR-3 oligomerization may provide an actin-independent mechanism to maintain distinct par protein domains in the early *Caenorhabditis elegans* embryo. *Biophys. J.* 101, 1412-1422. 10.1016/j.bpj.2011.07.030 21943422 PMC3177071

[JCS264093C11] Dechant, R. and Glotzer, M. (2003). Centrosome separation and central spindle assembly act in redundant pathways that regulate microtubule density and trigger cleavage furrow formation. *Dev. Cell* 4, 333-344. 10.1016/S1534-5807(03)00057-1 12636915

[JCS264093C12] Decker, M., Jaensch, S., Pozniakovsky, A., Zinke, A., O'Connell, K. F., Zachariae, W., Myers, E. and Hyman, A. A. (2011). Limiting amounts of centrosome material set centrosome size in *C. elegans* embryos. *Curr. Biol.* 21, 1259-1267. 10.1016/j.cub.2011.06.002 21802300

[JCS264093C13] Dewey, E. B., Taylor, D. T. and Johnston, C. A. (2015). Cell fate decision making through oriented cell division. *J. Dev. Biol.* 3, 129-157. 10.3390/jdb3040129 26844213 PMC4734650

[JCS264093C14] Glotzer, M. (2005). The molecular requirements for cytokinesis. *Science* 307, 1735-1739. 10.1126/science.1096896 15774750

[JCS264093C15] Gockenbach, M. S. (2006). *Understanding and Implementing the Finite Element Method*. SIAM.

[JCS264093C16] Goehring, N. W., Trong, P. K., Bois, J. S., Chowdhury, D., Nicola, E. M., Hyman, A. A. and Grill, S. W. (2011). Polarization of PAR proteins by advective triggering of a pattern-forming system. *Science* 334, 1137-1141. 10.1126/science.1208619 22021673

[JCS264093C17] Goldstein, B. and Hird, S. N. (1996). Specification of the anteroposterior axis in *Caenorhabditis elegans*. *Development* 122, 1467-1474. 10.1242/dev.122.5.1467 8625834

[JCS264093C18] Grill, S. W., Gönczy, P., Stelzer, E. H. K. and Hyman, A. A. (2001). Polarity controls forces governing asymmetric spindle positioning in the *Caenorhabditis elegans* embryo. *Nature* 409, 630-633. 10.1038/35054572 11214323

[JCS264093C19] Gross, P., Kumar, K. V., Goehring, N. W., Bois, J. S., Hoege, C., Jülicher, F. and Grill, S. W. (2019). Guiding self-organized pattern formation in cell polarity establishment. *Nat. Phys.* 15, 293-300. 10.1038/s41567-018-0358-7 31327978 PMC6640039

[JCS264093C20] Hannak, E., Kirkham, M., Hyman, A. A. and Oegema, K. (2001). Aurora-A kinase is required for centrosome maturation in *Caenorhabditis elegans*. *J. Cell Biol.* 155, 1109-1116. 10.1083/jcb.200108051 11748251 PMC2199344

[JCS264093C21] Hirani, N., Illukkumbura, R., Bland, T., Mathonnet, G., Suhner, D., Reymann, A.-C. and Goehring, N. W. (2019). Anterior-enriched filopodia create the appearance of asymmetric membrane microdomains in polarizing c. elegans zygotes. *J. Cell Sci.* 132, jcs230714. 10.1242/jcs.230714 31221727 PMC6679585

[JCS264093C22] Hundsdorfer, W. H., Verwer, J. G. and Hundsdorfer, W. (2003). *Numerical Solution of Timedependent Advection-Diffusion-Reaction Equations*, Vol. 33. Springer.

[JCS264093C23] Illukkumbura, R., Hirani, N., Borrego-Pinto, J., Bland, T., Ng, K. B., Hubatsch, L., McQuade, J., Endres, R. G. and Goehring, N. W. (2023). Design principles for selective polarization of PAR proteins by cortical flows. *J. Cell Biol.* 222, e202209111. 10.1083/jcb.202209111 37265444 PMC10238861

[JCS264093C24] Kapoor, S. and Kotak, S. (2019). Centrosome Aurora A regulates RhoGEF ECT-2 localisation and ensures a single PAR-2 polarity axis in *C. elegans* embryos. *Development* 146, dev174565. 10.1242/dev.174565 31636075 PMC7115938

[JCS264093C25] Klinkert, K., Levernier, N., Gross, P., Gentili, C., Von Tobel, L., Pierron, M., Busso, C., Herrman, S., Grill, S. W., Kruse, K. et al. (2019). Aurora A depletion reveals centrosome-independent polarization mechanism in *Caenorhabditis elegans*. *eLife* 8, e44552. 10.7554/eLife.44552 30801250 PMC6417861

[JCS264093C26] Kotak, S., Afshar, K., Busso, C. and Gönczy, P. (2016). Aurora A kinase regulates proper spindle positioning in *C. elegans* and in human cells. *J. Cell Sci.* 129, 3015-3025. 10.1242/jcs.184416 27335426 PMC6203311

[JCS264093C27] Kravtsova, N. and Dawes, A. T. (2014). Actomyosin regulation and symmetry breaking in a model of polarization in the early *Caenorhabditis elegans* embryo: symmetry breaking in cell polarization. *Bull. Math. Biol.* 76, 2426-2448. 10.1007/s11538-014-0016-x 25185748

[JCS264093C28] Ladurner, R., Kreidl, E., Ivanov, M. P., Ekker, H., Idarraga-Amado, M. H., Busslinger, G. A., Wutz, G., Cisneros, D. A. and Peters, J.-M. (2016). Sororin actively maintains sister chromatid cohesion. *EMBO J.* 35, 635-653. 10.15252/embj.201592532 26903600 PMC4801952

[JCS264093C29] Lang, C. F. and Munro, E. (2017). The PAR proteins: from molecular circuits to dynamic self-stabilizing cell polarity. *Development* 144, 3405-3416. 10.1242/dev.139063 28974638 PMC5665476

[JCS264093C30] Lang, C. F. and Munro, E. M. (2022). Oligomerization of peripheral membrane proteins provides tunable control of cell surface polarity. *Biophys. J.* 121, 4543-4559. 10.1016/j.bpj.2022.10.035 36815706 PMC9750853

[JCS264093C31] Lang, C. F., Maxian, O., Anneken, A. and Munro, E. (2024). Oligomerization and positive feedback on membrane recruitment encode dynamically stable PAR-3 asymmetries in the *C. elegans* zygote. *bioRxiv*. 10.1101/2023.08.04.552031 PMC13533420 41875854

[JCS264093C32] Longhini, K. M. and Glotzer, M. (2022). Aurora A and cortical flows promote polarization and cytokinesis by inducing asymmetric ECT-2 accumulation. *eLife* 11, e83992. 10.7554/eLife.83992 36533896 PMC9799973

[JCS264093C33] Loria, A., Longhini, K. M. and Glotzer, M. (2012). The RhoGAP domain of CYK-4 Has an essential role in RhoA activation. *Curr. Biol.* 22, 213-219. 10.1016/j.cub.2011.12.019 22226748 PMC3285270

[JCS264093C34] Maddox, A. S., Lewellyn, L., Desai, A. and Oegema, K. (2007). Anillin and the septins promote asymmetric ingression of the cytokinetic furrow. *Dev. Cell* 12, 827-835. 10.1016/j.devcel.2007.02.018 17488632

[JCS264093C35] Maddox, A. S., Azoury, J. and Dumont, J. (2012). Polar body cytokinesis. *Cytoskeleton* 69, 855-868. 10.1002/cm.21064 22927361

[JCS264093C36] Manzi, N. I., de Jesus, B. N., Shi, Y. and Dickinson, D. J. (2024). Temporally distinct roles of Aurora A in polarization of the *C. elegans* zygote. *Development* 151, dev202479. 10.1242/dev.202479 38488018 PMC11165718

[JCS264093C37] Mayer, M., Depken, M., Bois, J. S., Jülicher, F. and Grill, S. W. (2010). Anisotropies in cortical tension reveal the physical basis of polarizing cortical flows. *Nature* 467, 617-621. 10.1038/nature09376 20852613

[JCS264093C38] Michaud, A., Leda, M., Swider, Z. T., Kim, S., He, J., Landino, J., Valley, J. R., Huisken, J., Goryachev, A. B., von Dassow, G. et al. (2022). A versatile cortical pattern-forming circuit based on Rho, F-actin, Ect2, and RGA-3/4. *J. Cell Biol.* 221, e202203017. 10.1083/jcb.202203017 35708547 PMC9206115

[JCS264093C39] Michaux, J. B., Robin, F. B., McFadden, W. M. and Munro, E. M. (2018). Excitable RhoA dynamics drive pulsed contractions in the early *C. elegans* embryo. *J. Cell Biol.* 217, 4230-4252. 10.1083/jcb.201806161 30275107 PMC6279378

[JCS264093C40] Mogilner, A., Allard, J. and Wollman, R. (2012). Cell polarity: quantitative modeling as a tool in cell biology. *Science* 336, 175-179. 10.1126/science.1216380 22499937

[JCS264093C41] Motegi, F., Velarde, N. V., Piano, F. and Sugimoto, A. (2006). Two phases of astral microtubule activity during cytokinesis in *C. elegans* embryos. *Dev. Cell* 10, 509-520. 10.1016/j.devcel.2006.03.001 16580995

[JCS264093C42] Munro, E., Nance, J. and Priess, J. R. (2004). Cortical flows powered by asymmetrical contraction transport PAR proteins to establish and maintain anterior-posterior polarity in the early *C. elegans* embryo. *Dev. Cell* 7, 413-424. 10.1016/j.devcel.2004.08.001 15363415

[JCS264093C43] Nakayama, Y., Shivas, J. M., Poole, D. S., Squirrell, J. M., Kulkoski, J. M., Schleede, J. B. and Skop, A. R. (2009). Dynamin participates in the maintenance of anterior polarity in the *Caenorhabditis elegans* embryo. *Dev. Cell* 16, 889-900. 10.1016/j.devcel.2009.04.009 19531359 PMC2719978

[JCS264093C44] Nishikawa, M., Naganathan, S. R., Jülicher, F. and Grill, S. W. (2017). Controlling contractile instabilities in the actomyosin cortex. *eLife* 6, e19595. 10.7554/eLife.19595 28117665 PMC5354522

[JCS264093C45] Persson, P.-O. and Strang, G. (2004). A simple mesh generator in MATLAB. *SIAM Rev.* 46, 329-345. 10.1137/S0036144503429121

[JCS264093C46] Rappaport, R. (1985). Repeated furrow formation from a single mitotic apparatus in cylindrical sand dollar eggs. *J. Exp. Zool.* 234, 167-171. 10.1002/jez.1402340120 3989496

[JCS264093C47] Reymann, A.-C., Staniscia, F., Erzberger, A., Salbreux, G. and Grill, S. W. (2016). Cortical flow aligns actin filaments to form a furrow. *eLife* 5, e17807. 10.7554/eLife.17807 27719759 PMC5117871

[JCS264093C48] Robin, F. B., McFadden, W. M., Yao, B. and Munro, E. M. (2014). Single-molecule analysis of cell surface dynamics in Caenorhabditis elegans embryos. *Nat. Methods* 11, 677-682. 10.1038/nmeth.2928 24727651 PMC4046709

[JCS264093C49] Schaefer, M., Shevchenko, A. and Knoblich, J. A. (2000). A protein complex containing Inscuteable and the Galpha-binding protein Pins orients asymmetric cell divisions in Drosophila. *Curr. Biol.* 10, 353-362. 10.1016/S0960-9822(00)00401-2 10753746

[JCS264093C50] Scholze, M. J., Barbieux, K. S., De Simone, A., Boumasmoud, M., Süess, C. C. N., Wang, R. and Gönczy, P. (2018). Pi(4,5)p2 forms dynamic cortical structures and directs actin distribution as well as polarity in Caenorhabditis elegans embryos. *Development* 145, dev164988. 10.1242/dev.164988 29724757

[JCS264093C51] Schonegg, S., Constantinescu, A. T., Hoege, C. and Hyman, A. A. (2007). The Rho GTPase activating proteins RGA-3 and RGA-4 are required to set the initial size of PAR domains in *Caenorhabditis elegans* one-cell embryos. *Proc. Natl Acad. Sci. USA* 104, 14976-14981. 10.1073/pnas.0706941104 17848508 PMC1986598

[JCS264093C52] Severson, A. F. and Bowerman, B. (2003). Myosin and the PAR proteins polarize microfilament dependent forces that shape and position mitotic spindles in *Caenorhabditis elegans*. *J. Cell Biol.* 161, 21-26. 10.1083/jcb.200210171 12695495 PMC2172887

[JCS264093C53] Tame, M., Raaijmakers, J., Van Den Broek, B., Lindqvist, A., Jalink, K. and Medema, R. H. (2014). Astral microtubules control redistribution of dynein at the cell cortex to facilitate spindle positioning. *Cell Cycle* 13, 1162-1170. 10.4161/cc.28031 24553118 PMC4013166

[JCS264093C54] Tse, Y. C., Werner, M., Longhini, K. M., Labbe, J.-C., Goldstein, B. and Glotzer, M. (2012). RhoA activation during polarization and cytokinesis of the early *Caenorhabditis elegans* embryo is differentially dependent on NOP-1 and CYK-4. *Mol. Biol. Cell* 23, 4020-4031. 10.1091/mbc.e12-04-0268 22918944 PMC3469517

[JCS264093C55] Verbrugghe, K. J. C. and White, J. G. (2007). Cortical centralspindlin and G alpha have parallel roles in furrow initiation in early *C. elegans* embryos. *J. Cell Sci.* 120, 1772-1778. 10.1242/jcs.03447 17456550

[JCS264093C56] Werner, M., Munro, E. and Glotzer, M. (2007). Astral signals spatially bias cortical myosin recruitment to break symmetry and promote cytokinesis. *Curr. Biol.* 17, 1286-1297. 10.1016/j.cub.2007.06.070 17669650 PMC1978103

[JCS264093C57] Werner, M. E., Ray, D. D., Breen, C., Staddon, M. F., Jug, F., Banerjee, S. and Maddox, A. S. (2024). Mechanical and biochemical feedback combine to generate complex contractile oscillations in cytokinesis. *Curr. Biol.* 34, 3201-3214.e5. 10.1016/j.cub.2024.06.037 38991614 PMC11634113

[JCS264093C58] White, J. G. (1985). The astral relaxation theory of cytokinesis revisited. *BioEssays* 2, 267-272. 10.1002/bies.950020608

[JCS264093C59] White, J. G. and Borisy, G. G. (1983). On the mechanisms of cytokinesis in animal cells. *J. Theor. Biol.* 101, 289-316. 10.1016/0022-5193(83)90342-9 6683772

[JCS264093C60] Yao, B., Donoughe, S., Michaux, J. and Munro, E. (2022). Modulating RhoA effectors induces transitions to oscillatory and more wavelike RhoA dynamics in *Caenorhabditis elegans* zygotes. *Mol. Biol. Cell* 33, ar58. 10.1091/mbc.E21-11-0542 35138935 PMC9265151

[JCS264093C61] Zhao, P., Teng, X., Tantirimudalige, S. N., Nishikawa, M., Wohland, T., Toyama, Y. and Motegi, F. (2019). Aurora-A breaks symmetry in contractile actomyosin networks independently of its role in centrosome maturation. *Dev. Cell* 48, 631-645.e6. 10.1016/j.devcel.2019.02.012 30861375

