# Supplementary material for: A minimal mathematical model for polarity establishment and centralspindlin-independent cytokinesis
Source: J Cell Sci. 2025 Jun 11;138(11):jcs264093. doi: 10.1242/jcs.264093 (PMC12188313; doi:10.1242/jcs.264093)
Supplement: Supplementary information [file joces-138-264093-s1.pdf]

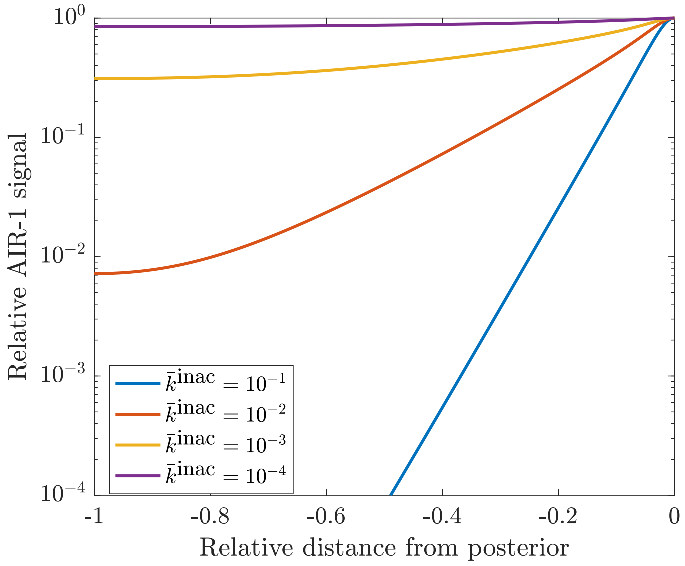

Fig. S1. AIR-1 signal vs. distance from posterior under polarization conditions (blue squares in Fig. 3(a)). We vary the level of phosphatase activity $\bar{k}^{\text{inac}}$ until the posterior level is less than 1% of the anterior level, settling on $\bar{k}^{\text{inac}} = 10^{-2}\ \mu m^{-2}$.

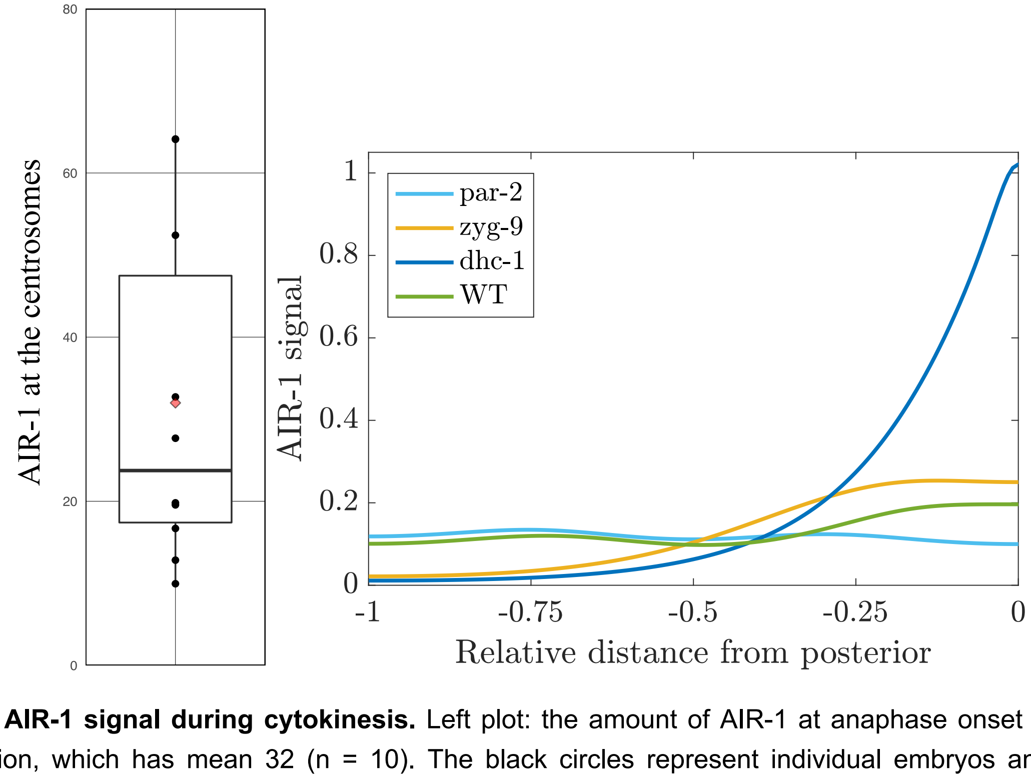

Fig. S2. AIR-1 signal during cytokinesis. Left plot: the amount of AIR-1 at anaphase onset relative to polarization, which has mean 32 (n = 10). The black circles represent individual embryos and the red diamond is the mean. Right plot: The corresponding AIR-1 profile on the embryo boundary, which comes from the solution of the diffusion equation (2) on the embryo cross section (see Fig. 2(b)).

Journal of Cell Science • Supplementary information

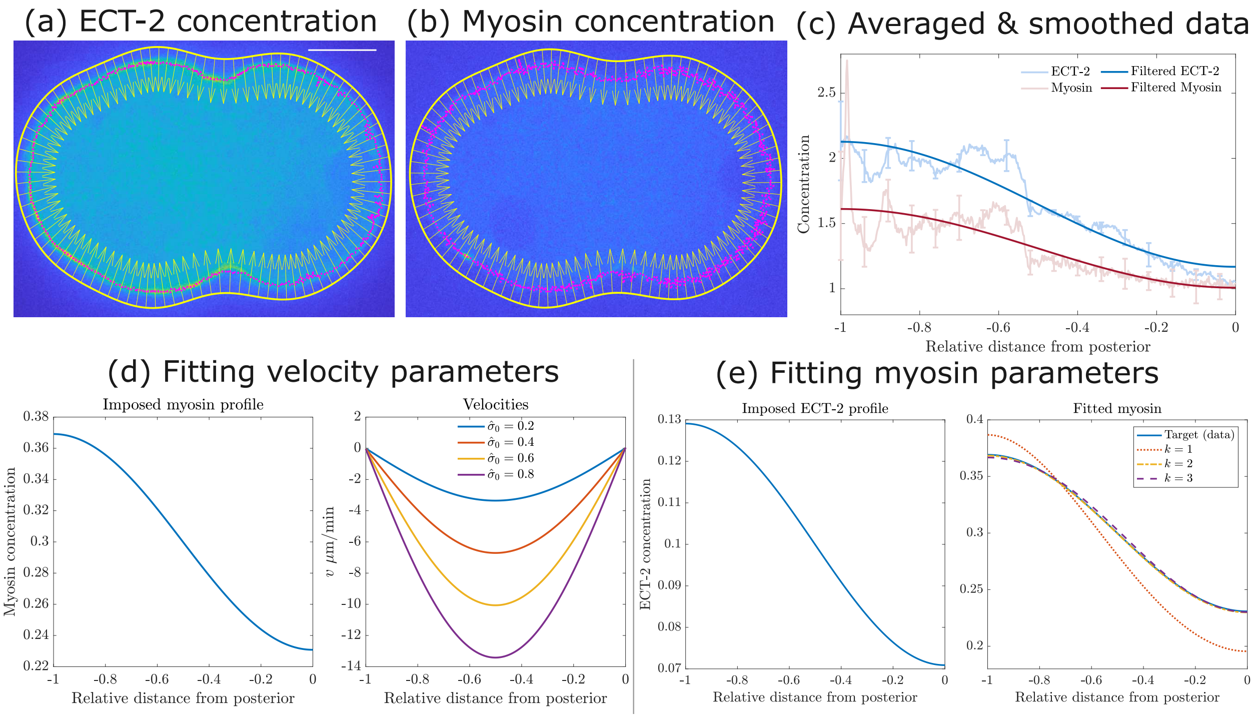

Fig. S3. Fitting myosin and flow parameters from experimental data in wild-type embryos. (a–c) We extract an ECT-2 and myosin concentration from N = 3 ECT-2 mNG and myosin mKate embryos imaged during pseudo-cleavage. (a,b) Show embryo images, where the scale bar is 10 μm, and (c) shows the resulting averaged and smoothed data over N = 3 embryos. (d–e) The ECT-2 and myosin profiles from the experimental data are used to obtain the parameters $\hat{\sigma}_0$, K_{EM}, and K_{fb} for each k.

Journal of Cell Science • Supplementary information

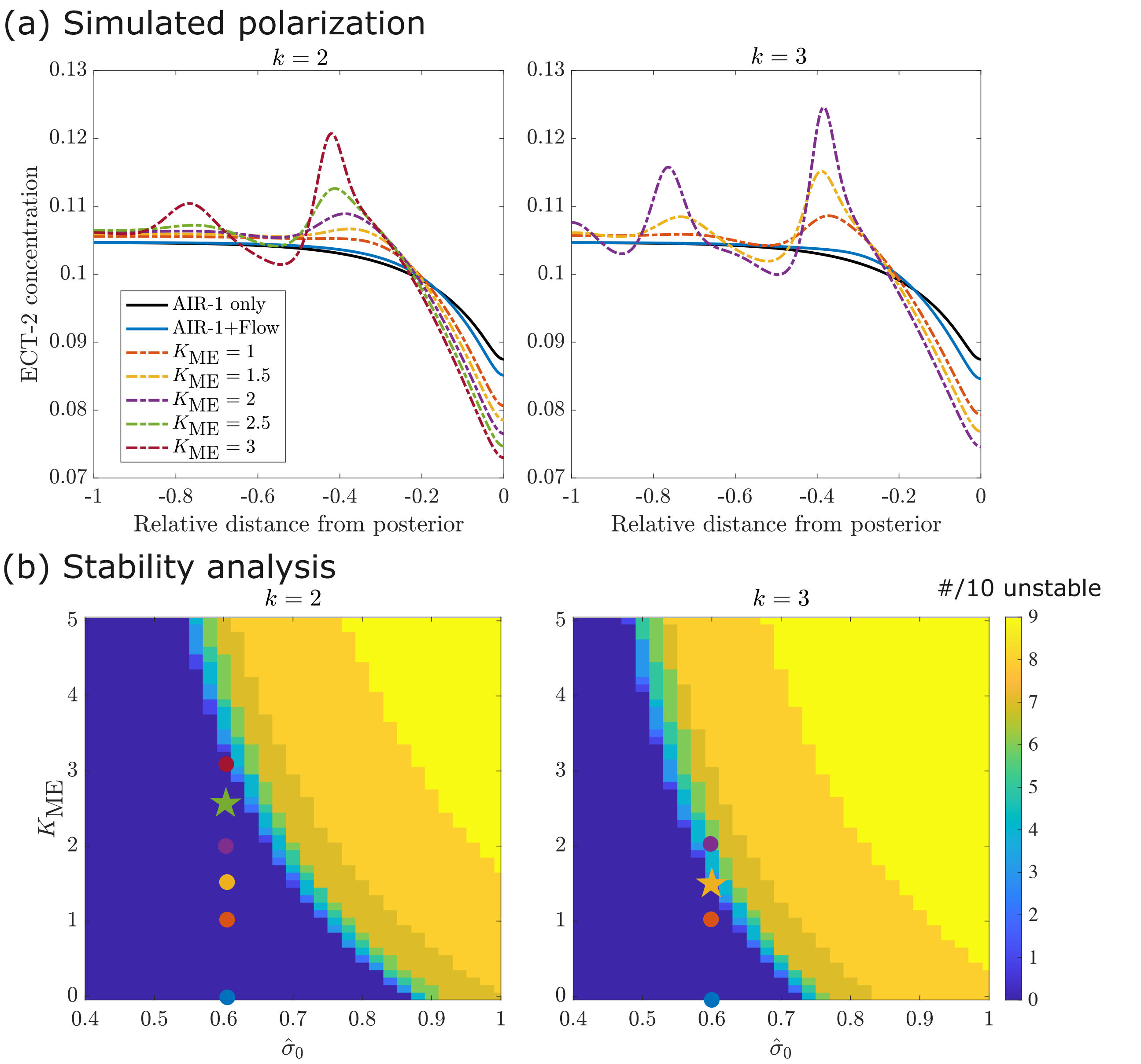

Fig. S4. Fitting the ECT-2 parameters, and relating those to the stability boundary. Top plots: for each k, we simulate polarization using the AIR-1 cue from centrosomes 1.9 μm away from the cortex. The black lines show the ECT-2 profile w ithout m yosin, w hile t he b lue l ines s how t he E CT-2 p rofile with advection only (no indirect recruitment). Other colored lines show the ECT-2 profile w ith a dvection and recruitment by myosin. Bottom plots: the stability diagram as a function of $\hat{\sigma}_0$ (flow strength) and K_{ME} (indirect recruitment strength). The chosen parameters (stars) lie on the edge of the unstable regime. In all cases, K_E^{on} is set so that 10% of ECT-2 is bound to the cortex.

Journal of Cell Science • Supplementary information

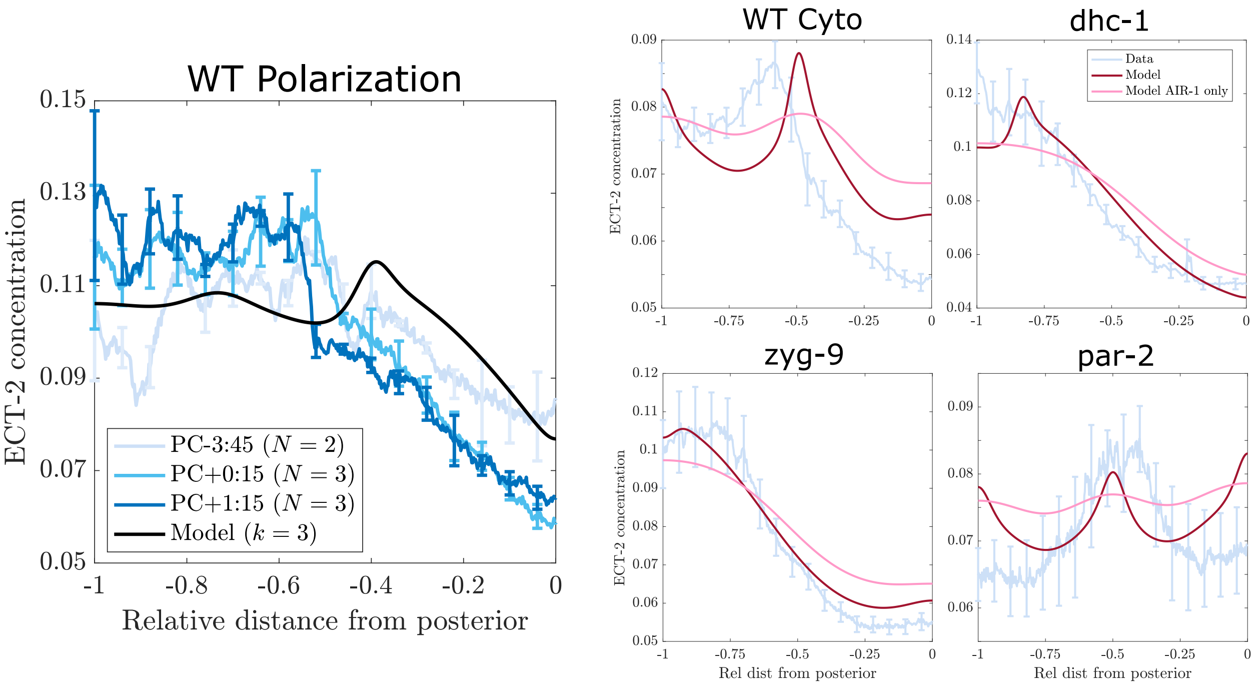

Fig. S5. Representative results when k = 3 in the myosin equation (1b). We show the resulting ECT-2 profiles in WT polarization (c.f. 3(d)) and cytokinesis (c.f. Fig. 5).

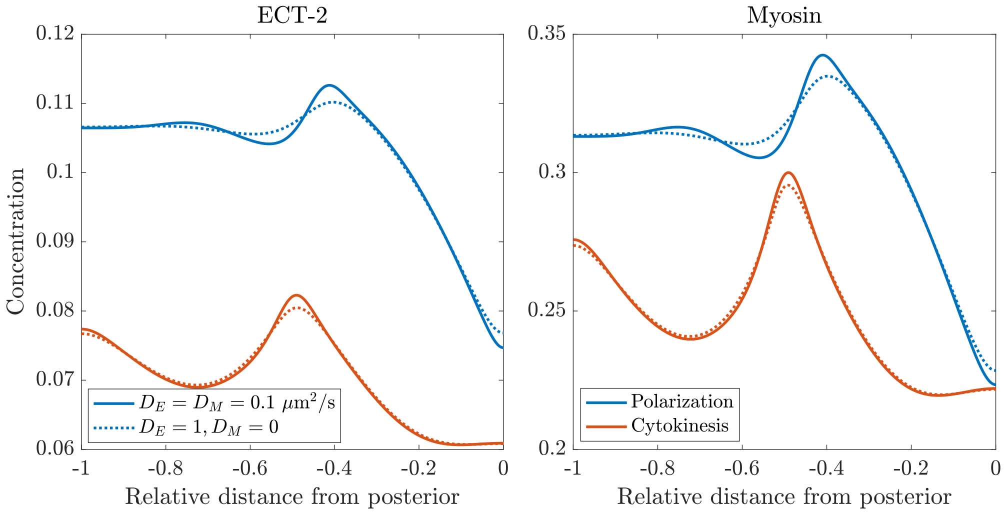

Fig. S6. Steady states for polarization and cytokinesis in wild-type embryos assuming (solid lines) $D_E = D_M = 0.1$ μm^2/s (control conditions), and (dotted lines) $D_E = 1$ μm^2/s and $D_M = 0$. Blue lines show polarization, red lines show cytokinesis.

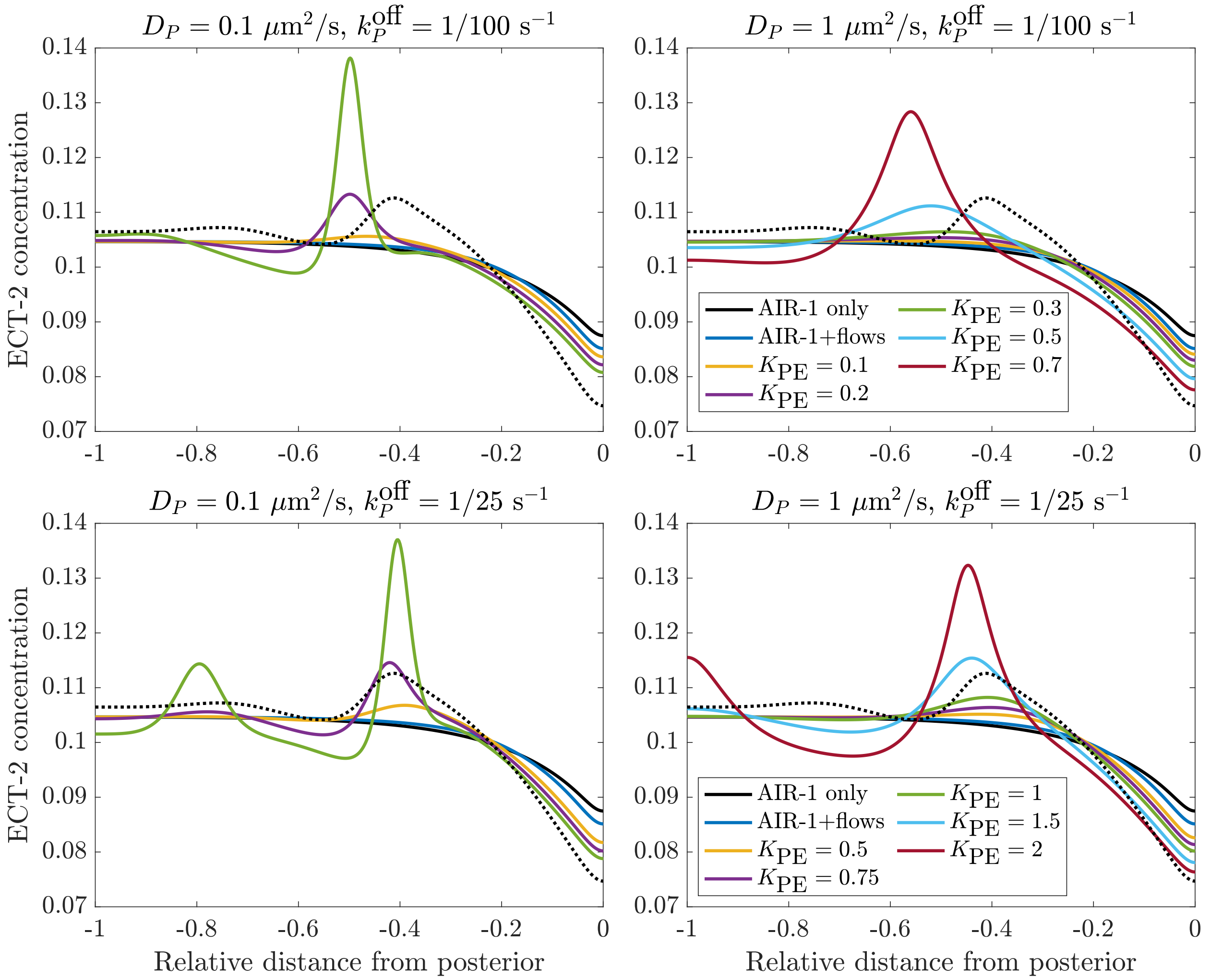

Fig. S7. Simulating polarization using the modified model (S2) where a longer-lived protein P recruits ECT-2. Solid lines show the results from the recruitment model (S2) with the indicated parameters, while the dotted black line is the steady state for the “minimal model” model (1) in the main text.

Journal of Cell Science • Supplementary information

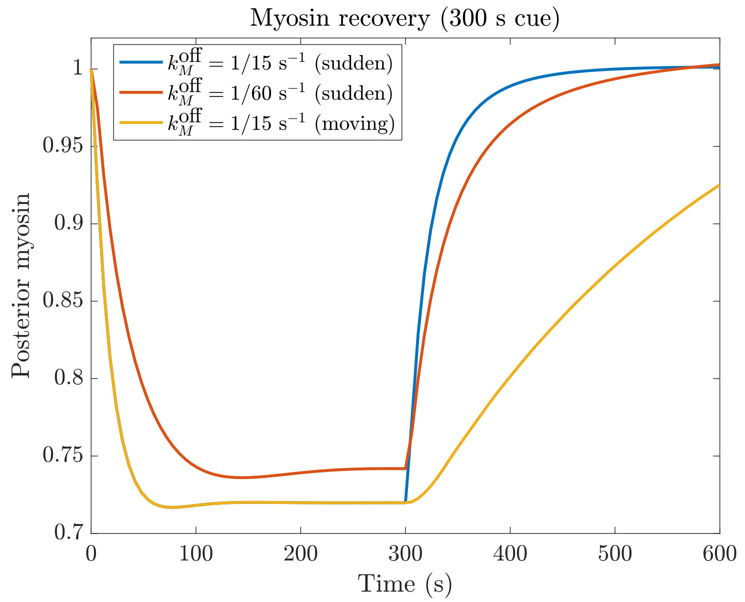

Fig. S8. Simulating myosin recovery during transient polarization. We show the myosin concentration at the posterior pole (normalized by the steady state) under three different conditions: control (blue, where the AIR-1 cue is present for 5 minutes), a simulation where the myosin lifetime is four times longer (red; to position the simulation at a similar point in the stability plane, we also modify the nonlinear negative feedback K_{fb} = 12), and a simulation where, starting at t = 5 mins, the centrosomes move at a linear rate from 2 to 10 µm from the cortex, with the corresponding change in the AIR-1 signal (see Fig. 3(b)) (yellow). While ECT-2 still responds rapidly to changes in the AIR-1 signal, the slow changes in the AIR-1 signal drive linear posterior recovery of ECT-2 (yellow curve), which accords better with the experimental data.

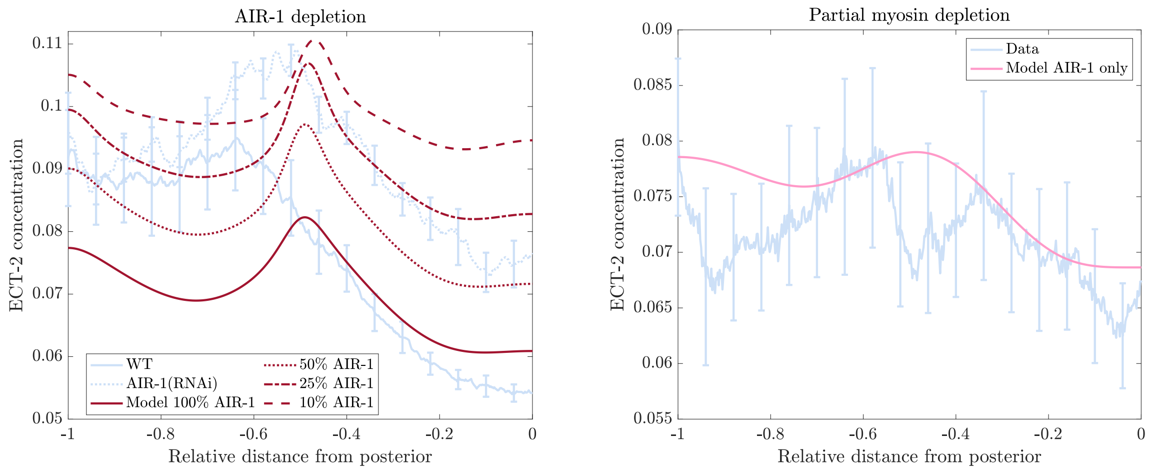

Fig. S9. Steady state ECT-2 accumulation during cytokinesis under AIR-1 and myosin depletion. Left plot: we show the steady state accumulation in the 50 s following cleavage ingression in control (wild type; N = 9) and AIR-1(RNAi) embryos (N = 11) (the constant of normalization is the same in both cases). We compare this to the model's simulated steady state using the wild-type AIR-1 profile shown in Fig. S2, multiplied by 1 (solid line), 0.5 (dotted line), 0.25 (dashed-dotted line), and 0.1 (dashed line). Right plot: we compare the AIR-1 only model prediction (pink line, same as the left panel of Fig. 5) to experimental data where myosin is partially depleted (N = 8; blue lines), again looking at the 50 s time interval that follows cleavage ingression.

Table S1. Relationship between dimensionless and dimensional parameters.

Dimensionless parameter	Dimensional expression	Description
$\hat{\sigma}_0$	$\sigma_0/(Lk_M^{\text{off}}\sqrt{\eta\gamma})$	Flow strength
$\hat{\ell}$	$\left(\sqrt{\eta/\gamma}\right)/L$	Hydrodynamic lengthscale
$\hat{D}_E$	$D_E/(L^2 k_M^{\text{off}})$	Diffusion of ECT-2
K_E^{on}	$k_E^{\text{on}}/(hk_M^{\text{off}})$	Binding rate of ECT-2
K_{ME}	$k_{\text{ME}}M^{(\text{Tot})}/k_E^{\text{on}}$	Relative recruitment ratio of ECT-2 by myosin
K_{AE}	k_{AE}	Inhibition of ECT-2 by AIR-1
K_E^{off}	$k_E^{\text{off}}/k_M^{\text{off}}$	ECT-2 unbinding ratio
$\hat{A}_{\text{sat}}$	$A_{\text{sat}}/A^{(\text{Tot})}$	AIR-1 saturation level
$\hat{D}_M$	$D_M/(L^2 k_M^{\text{off}})$	Diffusion of myosin
K_{EM}	$k_{\text{EM}}E^{(\text{Tot})}/(hk_M^{\text{off}})$	Activation of myosin by ECT-2 (through Rho)
K_{fb}	$k_{\text{fb}}\left(M^{(\text{Tot})}\right)^k/k_M^{\text{off}}$	Self-limiting myosin feedback

Journal of Cell Science • Supplementary information
