# Supplementary material for: A minimal mathematical model for polarity establishment and centralspindlin-independent cytokinesis
Source: J Cell Sci. 2025 Jun 11;138(11):jcs264093. doi: 10.1242/jcs.264093 (PMC12188313; doi:10.1242/jcs.264093)
Supplement: Supplementary file 1 [file joces-138-264093_review_history.pdf]

Journal of Cell Science

Review COMMONS

A minimal mathematical model for polarity establishment and centralspindlin-independent cytokinesis

Ondrej Maxian, Katrina M. Longhini and Michael Glotzer
DOI: 10.1242/jcs.264093

Editor: Renata Basto

Review timeline

Submission to Review Commons:	5 November 2024
Submission to Journal of Cell Science:	17 April 2025
Accepted:	27 April 2025

Reviewer 1

Evidence, reproducibility and clarity

Summary:

In this article, Maxian et al. propose a model combining 1-d simulations of ECT-2 and Myosin concentration at the cortex through binding/unbinding and advection at the cortex, with an input for AIR-1 cortical concentration based on the spatial localisation of the centrosomes in the cytoplasm. The objective of the authors is to recapitulate the role of (1) AIR-1, (2) its effector ECT-2 and (3) the downstream effector, driver of cortical flows, the molecular motors Myosin, in two key physiological processes, polarization and cell division. This is important as work over the last 10 years have emphasized the role of AIR-1 in embryo polarization. Previous biochemical-mechanical models have focused on RhoA/Myosin interactions (Nishikawa et al, 2017), the importance of a negative feedback and excitable RhoA dynamics (Michaux et al, 2018), or anterior PARs/posterior PARs/Myosin (Gross et al, 2019). The authors thus attempt to provide a new descriptive model in which RhoA is implicit, instead focusing on the role of centrosome localization on AIR-1 localization, and providing a framework to explore polarity establishment and cell division based on these 3 simple players.
The first part of the model is very reminiscent of previously published models, while the second instead provides a link between the initial polarizing cue AIR-1 and polarization. Based on this description, the model is precisely tuned to achieve polarization while matching experimental observations of flow speed and ECT-2 A/P enrichment shape. The results are therefore certainly new and interesting.

Major comments:

1. The authors use the position of the centrosomes as a static entry, resulting in a static AIR-1 input. Is this true, or are the positions of the centrosomes dynamically modulated over the course of the different processes simulated here (for example as a consequence of cortical flows?), and if so, is the assumption of immobile position?
2. While in its principle the model is quite simple and elegant, the detailed form of the equations describing the interactions between the players is more complex. Are all these required? If they are crucially important for the behavior of the model, these should be described more thoroughly, and if possible rooted more directly in experimental results, in particular:
- k(ME)*M*Ec (Linear enhancement term): why would myosin impact E concentration? The authors state, p.7, "There is a modest increase in the recruitment rate of ECT-2 due to cortical myosin (directly or indirectly), in a myosin concentration-dependent manner (Longhini and

© 2025. Published by The Company of Biologists under the terms of the Creative Commons Attribution License (https://creativecommons.org/licenses/by/4.0/).

Glotzer, 2022)." I could not find the data supporting this assumption - Longhini and Glotzer apparently rather point to a modulation of cortical flows. ("During anaphase, asymmetric ECT-2 accumulation is also myosin-dependent, presumably due to its role in generating cortical flows."). Embedding this effect in the recruitment rate instead of expecting it from the model thus appears awkward. Could the authors specify how they came to this conclusion, which the authors might have derived from observations made in their previous work, but maybe did not fully document there?
- k(EM)*E^2*Mc (ECT-2 non-linear impact on Myosin): does the specific of the value to convey the enhancement (square form) have an impact on the results?
- k(fb)*M^4 "The form of this term is a coarse-grained version of previously-published work (Michaux et al., 2018)." Myosin feedback on myosin localization proportionally to M^4 does not seem to directly derive from Michaux et al... Please detail this points more extensively and detail the derivation, in the supplements if not in the main text.
P23. Parameter values: "This is 1.5 times longer than the estimate for single molecules (Nishikawa et al., 2017; Gross et al., 2019) to reflect the more long-lived nature of myosin foci during establishment phase (Munro et al., 2004)." Not sure what the authors mean by more long-lived duration of foci during establishment phase. Seems rather arbitrary.
3. It would be very helpful (and indeed more convincing) to include a direct comparison between modeling results and experimental counterpart whenever possible. This might not be possible for some data (e.g. Fig. 3d from Cowan et al), but should be possible for other, in particular Fig. 3c and Fig. 5b, for the flow speed and ECT-2 profiles. In Fig. 5b in particular, previously published experimental data could be produced to give the reader to compare model with experiments (possibly provided as an inset, at least for the wild type conditions).

Minor comments

Fig. 5b: ECT-2 C 6A(dhc-1) do not seem to be referenced or discussed in the main text. Also, why present the results for the flow for 2 conditions and the ECT-2 localisation for 4? Or does the variation of ECT-2 not impact the flow profile?

p.6: Eqn 1a: ^ missing on 3rd E?

p.6: Given that the non-normalized data is used in the main text, and the normalized only appears in the supplemental, maybe star the dimensionless and remove all hats from the main for greater legibility?

p.14: replace "embryo treatment" with "experimental conditions"?

p.21, S4a: add A=Â/Atot

p.22: "L = 134.6 μm" - please write 134μm to retain the precision of original measurements

p.22: Please provide formula for all dimensionless values as a table at the end of the supplemental for the eager but less-mathematically proficient reader.

The authors' attention to providing *specific* citations including figure number corresponding to the specific point they reference in the papers they cite is appreciated

Significance

General assessment:

This modeling paper interestingly leverages existing experimental data to develop a new mathematical model of embryo polarization and cell division focusing on the role of AIR-1/Aurora Kinase. It combines classical 1-d advection/diffusion-reaction scheme with an upstream cue, AIR-1/Aurora Kinase, the profile of which is defined by the localization of the two centrosomes, and use the model as a framework to explore cortical flows and ECT-2 and Myosin cortical localization. Calibrated using information from polarization phase, the model recapitulates without any further tuning, in a variety of mutants, key localisation hallmarks of Ect-2 during cell division, simply

© 2025. Published by The Company of Biologists under the terms of the Creative Commons Attribution License (https://creativecommons.org/licenses/by/4.0/).

based on the localization of the centrosomes. Finally, it provides strong, experimentally testable predictions of the validity of the proposed model.

Advance:

In particular, this study provide compelling evidence showing that their model, based on dynamics during polarization, is sufficient to explain the ultra-sensitivity of cortical ECT-2 accumulation to centrosome distance during cell division. Their model further predicts that short ECT-2 cortical residence time is required to prevent advection-mediated counter-flows of ECT-2 that would otherwise prevent polarization, a prediction testable experimentally by engineering modifications of ECT-2 cortical residence time.

Audience:

This is primarily a modeling paper. Although the bulk of the article is written to capture the interest of cellular biologists with a sound backgrounds in mathematics and an interest in minimal models of cell division and polarization, the overall conclusions and prediction are further-reaching and would be of interest to a larger audience with an interest in cell division, polarization, and the role of Aurora Kinase in these processes.

Expertise:

Developmental biology / Cell biology / Biological physics

Reviewer 2

Evidence, reproducibility and clarity

The manuscript by Maxian, Longhini and Glotzer presents purely modeling work performed by the first author in conjunction with the already published experimental work by Longhini and Glotzer (eLife, 2022). The aim of the manuscript is to provide a mathematical model that connects the actomyosin contractility of the cell cortex in C. elegans zygote with the activity of the centrosomal kinase AurA (AIR-1 in C. elegans). The major claim of the authors is that their model, fitted to the experimental data pertaining to the zygote polarization, also describes dynamics during the zygote cytokinesis. In the model, the authors provide a heuristic approach to the biochemical dynamics, reducing their treatment to two variables: myosin and Ect2 Rho GEF. The biochemical model is integrated with a simple 1D active gel- type model for the cortical flow. The model uses static diffusive field of activity of AurA kinase in the cytoplasm as an input to their chemo-mechanical model.
Major concerns:
1. The biochemical model is highly heuristic and several major assumptions are poorly justified. Thus, the authors explicitly introduce recruitment of Ect2 by myosin, something apparently based on the experimental observations by Longhini and Glotzer in 2022, which had not been biochemically confirmed since with a clear molecular mechanism.
2. The contribution of AurA is introduced highly schematically as a term based on enzyme inhibition biochemistry that increases the off rate of Ect2. The major assumption of the model is that AurA phosphorylates Ect2 strictly on the membrane (cortex) of the cell. Why? No molecular justification is given. If the authors cannot provide clear justification, this major assumption has to be clearly declared as such. The phosphorylation/dephosphorylation dynamics of Ect2 is not considered at all.
3. In the equation for myosin, the authors introduce disassembly/ inactivation term proportional to the fourth order of concentration of myosin. Why? This is a major assumption, which appears to be derived from the work by Michaux et al. 2018. There the authors (Michaux et al.) postulated that the rate of inactivation of RhoA GTPase was somehow proportional to the fourth power of RhoA concentration. It appears that Maxian et al. further assume that the myosin concentration is fast variable enslaved by Rho, so that M ~ [RhoA]. They then presumably assume that if the rate of degradation/ inactivation of Rho is proportional to the forth power of Rho concentration, so is true for myosin (M). This is a logical error and is not justified. An important question, why do the current authors need this unusual assumption with such a high power of M disassembly/inactivation? Perhaps, this is because without this rather dubious term the cortex

© 2025. Published by The Company of Biologists under the terms of the Creative Commons Attribution License (https://creativecommons.org/licenses/by/4.0/).

flow produces a blow-up of myosin concentration? This would be expected in their mechanical model - the continuous flow of actomyosin not compensated by cortex disassembly generally causes blow-up of biochemical concentrations transported by the flow, this is a known problem of the "simple" active gel model used by the authors. Maxian et al. have to provide clear derivation of the term -kfb*M^4 and also demonstrate why they need this exotic assumption.
4. The equation for myosin M has a membrane-binding term, which is second order in concentration of Ect2 ~E^2, without which the model will not show the instability that the authors need. The only justification given is that "some nonlinearity is required". A proper derivation should be given here.
5. The diffusion coefficients for Ect2 and myosin are chosen to be the same. Why? Clearly these molecules so different in size - myosin being a gigantic cluster monster of ~300 nm believed to be bound to actin, should have a much smaller diffusion coefficient?
6. There are confusing statements regarding the role of actomyosin flows. In the beginning of the manuscript, the authors seem to state that since Ect2 has a high off rate, the effect of the flow on Ect2 localization is negligible in comparison with direct binding to myosin. Later, the authors state that flows are absolutely essential for the patterning. The authors need to clearly explain where and how the flows are important or not.
Minor points:
1. page 9. Why is the rate of dephosphorylation of AurA is named Koff?
2. page 10. "Note that the model is calibrated to predict... which matches experimental observations" - this sentence needs changing. You want to say that you fit the model to experiments in the Longhini and Glotzer paper. There is no prediction here.
3. page 14. "A plot of Ect-2 accumulation as a function of distance from the nearest cortex..." - clearly the word "centrosome" is meant here instead of "cortex".
4. page 16. "Inactive, non-phosphorylatable version of Ect-2..." - non-phosphorylatable is clear, but why inactive?

Significance

This reviewer sees limited significance of this manuscript to the field in general. The modeling approach is hardly novel as it is based on a variety of published models, all cited by the authors, to be precise. The model, being very simplistic and heuristic, is not predictive. The main novelty of the current manuscript is the introduction of the effect of Aurora A on the activity of the actomyosin cortex. Since this is taken to be very schematic, simply via the effective increase in the off rate of Ect2, the model is showing that it is consistent with the earlier published experimental results by Longhini and Glotzer. This is to be expected. The main claim of the authors, that the model fitted to the polarization data also qualitatively describes the cytokinesis (there are no quantitative data to compare to) is probably valid, but the result is not surprising either. At best, the model can be labeled as fitted to the data and confirming the experimental results. Since it contains several postulated heuristic terms not properly justified on the mechanistic level, this is also not surprising.

Reviewer 3

Evidence, reproducibility and clarity

Maxian et al. developed a mathematical model to explain the essential elements and interactions necessary and sufficient for the polarisation of the C. elegans zygote. The initiation of zygote polarisation has been extensively studied in recent years, highlighting the role of the centrosomal kinase Aurora-A (AIR-1) in controlling the cortical distribution of RhoGEF (ECT-2) and actomyosin contractility during polarisation. Although genetic experiments have demonstrated their function in this process, it remains to be tested whether these factors and their interactions are sufficient to induce polarisation.

This work has provided a theoretical framework to predict the activity of AIR-1 in the cytoplasm and at the cell cortex, and the cortical distribution of ECT-2 and myosin-II (NMY-2). This framework can recapitulate the dynamic rearrangement of ECT-2 and myosin-II during polarisation, with centrosomes positioned at the posterior pole of the zygote. This model can explain, at least in part, the asymmetric distribution of ECT-2 and myosin-II in the zygote undergoing cytokinesis, suggesting that the mechanism of AIR-1-mediated control of ECT-2 and

© 2025. Published by The Company of Biologists under the terms of the Creative Commons Attribution License (https://creativecommons.org/licenses/by/4.0/).

myosin-II would regulate patterning during polarisation and cytokinesis. This theoretical framework is developed with reasonable assumptions based on previous genetic experiments (except for the myosin-dependent regulation of ECT-2; see comments below).

Issue #1

The authors insist that this model correctly predicts the spatio-temporal dynamics of ECT-2 and myosin-II during polarisation and cytokinesis. However, the predicted results do not reproduce the in vivo pattern of ECT-2 in both phases. ECT-2 is cleared from the posterior cortex and establishes a graded pattern across the antero-posterior axis during polarisation (see their previous publication in eLife 2022, 11, e83992, Fig1A -480s) and cytokinesis (see eLife 2022, 11, e83992, Fig1C 60s and 120s). During both stages, ECT-2 does not show local enrichment at the boundary between the anterior and posterior cortical domains in vivo. In fact, when comparing the predicted results with the in vivo pattern of ECT-2 and cortical flow, the authors used non-quantitative descriptions such as 'in good agreement', 'a realistic magnitude', 'resemble'. These vague descriptions should be revised and a quantitative assessment of ECT-2 distribution between in silico and in vivo should be included in a revised manuscript.

Issue #2

I assume that the strange local enrichment of ECT-2 at the anteroposterior boundary is due to their assumption that the binding rate of ECT-2 is increased by a linear increase via cortical myosin-II (page 6). This assumption is not directly supported by experimental evidence. A previous study by the same group (eLife 2022, 11, e83992) showed that a progressive increase in ECT-2 concentration at the anterior cortex is partially accompanied by an increase in cortical flow and transport of myosin-II from the posterior pole to the anterior cortex. This observation supports the idea that ECT-2 may associate with cortical components transported by myosin-II based cortical flow. This unrealistic assumption makes the predicted distribution pattern of ECT-2 almost identical to that of cortical myosin-II, resulting in an increase in the concentration of ECT-2 at the anteroposterior boundary where myosin-II forms pheudo-cleavages and cleavage furrows. The authors should clarify why their mathematical model used this assumption and provide a comprehensive analysis and evaluation of the parameter value for an ECT-2-myosin-II interaction.

Significance

This work includes a valuable tool that can be used to explain other actomyosin-mediated polarisation processes. Although the paper provides useful insights in principle, the weakness of this work is that the model is designed with parameter sets that only recapitulate previously published phenotypes. Therefore, this paper confirms previous findings and provides less/no new mechanistic insights into cell polarisation. As such, this work would be of interest to specialised cell biologists and biophysicists working on the cytoskeleton and cell division, but will not be of general interest to biologists and biochemists.

Author response to reviewers' comments

Manuscript number: RC-2024-02773

Corresponding Author: Glotzer, Michael

1 Summary of changes

There were several points that were raised by multiple reviewers, which we respond to as follows.

1. The reviewers pointed to a lack of clear comparison with experimental data. Perhaps this was insufficiently clear in the first submission, but the analysis of ECT-2 localization during cytokinesis was intended as a validation of the model, parameterized based on polarization and applied without further modification to cytokinesis. These situations differ in

© 2025. Published by The Company of Biologists under the terms of the Creative Commons Attribution License (https://creativecommons.org/licenses/by/4.0/).

numerous respects: centrosome number, centrosome size, and we used several experimental conditions to control centrosome positioning. To address this more extensively, in the revised submission we analyzed our data further (Longhini and Glotzer, 2022) to extract profiles of ECT-2 and myosin. We used these profiles both to constrain model parameters (Appendix B.3) and to compare with model predictions for both polarization and cytokinesis (Figs. 3 and 5).

2. All of the reviewers pointed to our assumption that myosin indirectly recruits ECT-2. We apologize for a lack of clarity in the original draft about this. We had intended to convey the hypothesis that ECT-2 is recruited by a species that is advected with myosin, but for the sake of the minimal model we do not introduce any extra equations for this species and instead assume it colocalizes with myosin. In the revised manuscript, we address this by clearly listing the assumption (#2 on p. 7), and by comparing to an alternative model (Eq. (S4) and Fig. S7) that accounts directly for a third advected species. We also document specifically (second panel from left in Fig. 4) why the short residence time of ECT-2 makes patterning by pure advection impossible. That said, we still do not know the identity of this factor.

3. The reviewers pointed out that our use of the M^4 term to limit contractility was dubious. This was a (probably misguided) attempt to use previously-published models to constrain our model. In the revised submission, we replaced this term with a more general nonlinear term M^k, where we first demonstrate that $k = 1$ is insufficient to match the data (p. 32), then the corresponding results for $k = 3$ are not very different. Put another way, we empirically demonstrate that the specific form of this nonlinear term is not important, as long as it prevents contractile instabilities (as pointed out by one of the reviewers).

4. Apparently, our extension of the model to cytokinesis, and the evidence for validation of the model, was not clear in the original draft. Because of this, we reformulated the section (3.4) and figure (5) on cytokinesis. We identified four representative examples of centrosome positions, then compared the experimental profile of ECT-2 accumulation to the model result. For simplicity, we also eliminated the simulations of the non-phosphorylatable inactive copy of ECT-2 ("ECT-2 6A"). A more detailed analysis of that data revealed that the pattern of accumulation of ECT-2 6A at cleavage furrowing was more similar to the end of polarization, indicating that this copy of ECT-2 appears to have much slower turnover than the endogenous copy (as would expected from phosphorylation-dependent membrane displacement).

5. Fundamentally, our study addresses a similar question to (Illukkumbura et al., 2023), in the sense that we seek to understand how cortical flows could pattern ECT-2 and myosin, even though the residence time of ECT-2 is very low. Despite the similarities, it differs from the cited study in that ECT-2 is not an inert component that is asymmetrically distributed, but rather a component which regulates myosin levels and cortical flows, ultimately feeding back on its own accumulation. Due to these similarities and differences, we added an expository section in the discussion (p. 18) comparing our results to those of that study.

2 Point-by-point description of the revisions

Reviewer 1

In this article, Maxian et al. propose a model combining 1-d simulations of ECT-2 and Myosin concentration at the cortex through binding/unbinding and advection at the cortex, with an input for AIR-1 cortical concentration based on the spatial localisation of the centrosomes in the cytoplasm. The objective of the authors is to recapitulate the role of (1) AIR-1, (2) its effector ECT-2 and (3) the downstream effector, driver of cortical flows, the molecular motors Myosin, in two key physiological processes, polarization and cell division. This is important as work over the last 10 years have emphasized the role of AIR-1 in embryo polarization. Previous biochemical-mechanical negative feedback and excitable RhoA dynamics (Michaux et al, 2018), or anterior PARs/posterior PARs/Myosin (Gross et al, 2019). The authors thus attempt to provide a new descriptive model in which RhoA is implicit, instead focusing on the role of centrosome localization on AIR-1 localization, and providing a framework to explore polarity establishment and cell division

© 2025. Published by The Company of Biologists under the terms of the Creative Commons Attribution License (https://creativecommons.org/licenses/by/4.0/).

based on these 3 simple players. The first part of the model is very reminiscent of previously published models, while the second instead provides a link between the initial polarizing cue AIR-1 and polarization. Based on this description, the model is precisely tuned to achieve polarization while matching experimental observations of flow speed and ECT-2 A/P enrichment shape. The results are therefore certainly new and interesting.

Thank you for the positive assessment!

Major comments:

1. *The authors use the position of the centrosomes as a static entry, resulting in a static AIR-1 input. Is this true, or are the positions of the centrosomes dynamically modulated over the course of the different processes simulated here (for example as a consequence of cortical flows?), and if so, is the assumption of immobile position?*

   We assume that the centrosomes are fixed on the timescale of the cortical dynamics, and study how the cortex responds to a static AIR-1 signal (see clarifying comment on p. 4). In Fig. S4, we show that the cortex responds rapidly to changes in the existence or position of the AIR-1 signal. As such, slower dynamics might be the result of slowly moving centrosomes, as we show in supplementary simulations (Fig. S8).

2. *While in its principle the model is quite simple and elegant, the detailed form of the equations describing the interactions between the players is more complex. Are all these required? If they are crucially important for the behavior of the model, these should be described more thoroughly, and if possible rooted more directly in experimental results:*

   Thank you for this comment. We agree that there were several non-trivial terms in our “minimal” model. Our guiding principle for the revision was to reduce complexity and better justify the terms that are included.

   (a) $k_{ME}M\,E_c$ *(Linear enhancement term): why would myosin impact E concentration? The authors state, p.7, ”There is a modest increase in the recruitment rate of ECT-2 due (Longhini and Glotzer, 2022).” I could not find the data supporting this assumption - Longhini and Glotzer apparently rather point to a modulation of cortical flows. (”During anaphase, asymmetric ECT-2 accumulation is also myosin-dependent, presumably due to its role in generating cortical flows.”). Embedding this effect in the recruitment rate instead of expecting it from the model thus appears awkward. Could the authors specify how they came to this conclusion, which the authors might have derived from observations made in their previous work, but maybe did not fully document there?*

   This is an important issue. Since it was raised by all of the reviewers, we addressed it on page 1 above. Throughout the manuscript (Figs. 4 and S4), we tried to highlight that cortical flows are insufficient to localize ECT-2, while the recruitment hypothesis provides a better match to the experimental data. The recruitment by an advected species was speculated upon in Longhini and Glotzer: ”Rather, we favor a model in which the association of ECT-2 with the cortex involves interactions with cortical component(s) that are concentrated by cortical flows.”

   (b) $k_{EM}E^2M_c$ *(ECT-2 non-linear impact on Myosin): does the specific form of the value to convey the enhancement (square form) have an impact on the results?*

   The specific form does not have an impact. In fact, in the revised version, our experimental data shows an asymmetry in myosin that is actually *lower* than ECT-2. As such, a nonlinear term here lacks justification, and we switched to a linear term of the form $k_{EM}EM_c$ (see model equations on p. 6).

   (c) $K_{fb}M^4$ *”The form of this term is a coarse-grained version of previously-published work*

   (Michaux et al., 2018).” Myosin feedback on myosin localization proportionally to M^4 *does not seem to directly derive from Michaux et al. Please detail this points more extensively and detail the derivation, in the supplements if not in the main text.*

   Based on this comment and that of reviewer 2, we decided to switch to a more general term for nonlinear negative feedback, as discussed in point 3 on page 1.

   (d) *P23. Parameter values: ”This is 1.5 times longer than the estimate for single molecules*

© 2025. Published by The Company of Biologists under the terms of the Creative Commons Attribution License (https://creativecommons.org/licenses/by/4.0/).

(Nishikawa et al., 2017; Gross et al., 2019) to reflect the more long-lived nature of myosin foci during establishment phase (Munro et al., 2004)." Not sure what the authors mean by more long-lived duration of foci during establishment phase. Seems rather arbitrary.
This was a misstatement on our part. A closer look at Gross et al. revealed that, under conditions similar to those we simulate (initial polarity establishment), the residence time of myosin is about 15 s (off rate 0.06 s^{-1}). We modified our justification (p. 30) to include this. We also looked at the effect of longer myosin residence time on polarity establishment (Fig. S8).

3. *It would be very helpful (and indeed more convincing) to include a direct comparison between modeling results and experimental counterpart whenever possible. This might not be possible for some data (e.g. Fig. 3d from Cowan et al), but should be possible for other, in particular Fig. 3c and Fig. 5b, for the flow speed and ECT-2 profiles. In Fig. 5b in particular, previ- ously published experimental data could be produced to give the reader to compare model with experiments (possibly provided as an inset, at least for the wild type conditions).*
We tried to bring in more data based on what was available from previous work (Longhini and Glotzer, 2022). Frame intervals of 10 s prohibited a PIV analysis for flow speeds, and punctate myosin profiles often made it difficult to measure myosin concentration. We were, however, able to extract the ECT-2 concentration from our previous movies and compare it to the model results. We included these comparisons in Figs. 3 and 5, with accompanying discussion in the text.

Minor comments:

1. *Fig. 5b: ECT-2 C 6A(dhc-1) do not seem to be referenced or discussed in the main text.*

   Also, why present the results for the flow for 2 conditions and the ECT-2 localisation for 4? Or does the variation of ECT-2 not impact the flow profile?
   As discussed above on p. 2 (#4), we decided to reformulate the cytokinesis figure to incorporate more experimental data. Since we have detailed data on ECT-2 localization, we presented these in Fig. 5 for four experimental conditions, comparing each to the model.

2. *p.6: Given that the non-normalized data is used in the main text, and the normalized only appears in the supplemental, maybe star the dimensionless and remove all hats from the main for greater legibility?*
   We changed the notation to make the main text variables (dimensional) unadorned, while the dimensionless variables in the SI now have hats.

3. *p.6: Eqn 1a: carrot missing on 3rd E?*
   This is now a moot point because of the previous comment.

4. *p.14: replace "embryo treatment" with "experimental conditions"?*

   We changed "embryo treatment" to "experimental conditions" globally.

5. *p.21, S4a: add* $A = \hat{A} / A^{(Tot)}$

   We added it in the last display on p. 28.

6. *p.22: "L = 134.6 µm" - please write 134 µm to retain the precision of original measurements*

   We made this change.

7. *p.22: Please provide formula for all dimensionless values as a table at the end of the*

© 2025. Published by The Company of Biologists under the terms of the Creative Commons Attribution License (https://creativecommons.org/licenses/by/4.0/).

supple- mental for the eager but less-mathematically proficient reader.
We added Table 1 to list the relationship between dimensional and dimensionless parameters.

Reviewer 2

The manuscript by Maxian, Longhini and Glotzer presents purely modeling work performed by the first author in conjunction with the already published experimental work by Longhini and Glotzer (eLife, 2022). The aim of the manuscript is to provide a mathematical model that connects the actomyosin contractility of the cell cortex in C. elegans zygote with the activity of the centrosomal kinase AurA (AIR-1 in C. elegans). The major claim of the authors is that their model, fitted to the experimental data pertaining to the zygote polarization, also describes dynamics during the zygote cytokinesis. In the model, the authors provide a heuristic approach to the biochemical dynamics, reducing their treatment to two variables: myosin and Ect2 Rho GEF. The biochemical model is integrated with a simple 1D active gel-type model for the cortical flow. The model uses static diffusive field of activity of AurA kinase in the cytoplasm as an input to their chemo-mechanical model.

Major concerns:

1. *The biochemical model is highly heuristic and several major assumptions are poorly justified.*

   Thus, the authors explicitly introduce recruitment of Ect2 by myosin, something apparently based on the experimental observations by Longhini and Glotzer in 2022, which had not been biochemically confirmed since with a clear molecular mechanism.

   This is an important issue, and we appreciate your concern which was shared by the other reviewers. As discussed above on p. 1, we tried to justify this assumption better by (a) clearly stating it on p. 7, and (b) demonstrating that the dynamics we observe in live embryos are impossible without it. The model confirms what was pointed out by Longhini and Glotzer, that the short residence time of ECT-2, combined with *in vivo* flow speeds on the order of 10 μm/min, make it impossible for cortical flows alone to redistribute ECT-2.

2. *The contribution of AurA is introduced highly schematically as a term based on enzyme in- hibition biochemistry that increases the off rate of Ect2. The major assumption of the model is that AurA phosphorylates Ect2 strictly on the membrane (cortex) of the cell. Why? No molecular justification is given. If the authors cannot provide clear justification, this ma- jor assumption has to be clearly declared as such. The phosphorylation/dephosphorylation dynamics of Ect2 is not considered at all.*
   We clarified that the species we consider in the model (E) is unphosphorylated ECT-2, so that the negative flux comes from either unbinding or phosphorylation. Of course, AIR-1 phosphorylates ECT-2 in the cytoplasm as well, but our model only tracks the binding of *unphosphorylated* ECT-2 to the cortex. We clarified this on p. 6.
3. *In the equation for myosin, the authors introduce disassembly/ inactivation term proportional to the fourth order of concentration of myosin. Why? This is a major assumption, which appears to be derived from the work by Michaux et al. 2018. There the authors (Michaux et al.) postulated that the rate of inactivation of RhoA GTPase was somehow proportional to the fourth power of RhoA concentration. It appears that Maxian et al. further assume that the myosin concentration is fast variable enslaved by Rho, so that M ~ [RhoA]. They then presumably assume that if the rate of degradation/ inactivation of Rho is proportional to the fourth power of Rho concentration, so is true for myosin (M). This is a logical error and is not justified. An important question, why do the current authors need this unusual assumption with such a high power of M disassembly/inactivation? Perhaps, this is because without this rather dubious term the cortex flow produces a blow-up of myosin concentration? This would be expected in their mechanical model - the continuous flow of actomyosin not compensated by cortex disassembly generally causes blow-up of biochemical concentrations transported by the flow, this is a known problem of the "simple" active gel model used by the authors. Maxian et al. have to provide clear derivation of the term* $-K_{fb}M^4$ *and also demonstrate why they need this exotic assumption.*

   As mentioned above on p. 1, this was a misguided attempt on our part to use previous litera-

© 2025. Published by The Company of Biologists under the terms of the Creative Commons Attribution License (https://creativecommons.org/licenses/by/4.0/).

ture to directly assign values to model parameters. In the revised manuscript, we considered a more general term for the nonlinear feedback. The fitting occurs in Fig. S3, where we impose the ECT-2 profile during pseudo-cleavage and try to fit the myosin profile. $k = 1$ is eliminated because the ECT-2 and myosin have different asymmetries. Higher order nonlinearities ($k = 2, 3$) are successful in fitting the experimental data. In the main text, we present results from $k = 2$, then use Fig. S5 to present results on the $k = 3$ case.

4. *The equation for myosin M has a membrane-binding term, which is second order in concentration of Ect2 $\sim E^2$, without which the model will not show the instability that the authors need. The only justification given is that "some nonlinearity is required". A proper derivation should be given here.*

   Our experimental data shows an asymmetry in myosin that is actually *lower* than ECT-2. As such, a nonlinear term in the binding rate lacks justification, and we switched to a linear term of the form $k_{EM}EM_C$ (see model equations on p. 6).

5. *The diffusion coefficients for Ect2 and myosin are chosen to be the same. Why? Clearly these molecules so different in size - myosin being a gigantic cluster monster of $\sim$ 300nm believed to be bound to actin, should have a much smaller diffusion coefficient?*

   Thank you for raising this point. We used the same diffusion coefficient for simplicity; because its dimensionless value is less than 10^{-4}, diffusion is relatively unimportant in shaping the concentration fields. If we assume instead, for instance, that myosin cannot diffuse in the membrane, while ECT-2 has a ten-fold larger diffusion coefficient, the steady state profiles of ECT-2 and myosin are changed by at most 5% (see Fig. S6).

6. *There are confusing statements regarding the role of actomyosin flows. In the beginning of the manuscript, the authors seem to state that since Ect2 has a high off rate, the effect of the flow on Ect2 localization is negligible in comparison with direct binding to myosin. Later, the authors state that flows are absolutely essential for the patterning. The authors need to clearly explain where and how the flows are important or not.*
   Thank you for pointing out this confusion. In the revised manuscript, we tried to be explicit that the combination of recruitment and flows is essential for patterning ECT-2. We did this in Figs. 4 and 5 by showing the results of simulations without recruitment (Fig. 4) and without recruitment and flows (Fig. 5).

Minor points:

1. *page 9. Why is the rate of dephosphorylation of AurA is named Koff?*

   We changed the notation to $\bar{k}^{\text{inac}}$ to reflect inactivation.

2. *page 10. "Note that the model is calibrated to predict... which matches experimental observa- tions" - this sentence needs changing. You want to say that you fit the model to experiments in the Longhini and Glotzer paper. There is no prediction here.*
   We removed this sentence.

3. *page 14. "A plot of Ect-2 accumulation as a function of distance from the nearest cortex..."*

   - clearly the word "centrosome" is meant here instead of "cortex".

   What was meant by this sentence was the distance from the centrosome to the nearest cortex pole (anterior or posterior). We modified it to make this more clear (p. 15).

4. *page 16. "Inactive, non-phosphorylatable version of Ect-2..." - non-phosphorylatable is clear, but why inactive?*
   As discussed on p. 2 (#4), we decided to simplify the cytokinesis figure and remove the

© 2025. Published by The Company of Biologists under the terms of the Creative Commons Attribution License (https://creativecommons.org/licenses/by/4.0/).

simulations with non-phosphorylatable ECT-2. While it is not relevant, the ECT-2 6A variant represents a fragment of the protein that lacks the catalytic domain. Our original goal was to use these data to track the ECT-2 localization without perturbing the system biochemistry, but the data gave the hint of longer exchange kinetics, which confounded our analysis.

Reviewer 3

Maxian et al. developed a mathematical model to explain the essential elements and interactions necessary and sufficient for the polarisation of the C. elegans zygote. The initiation of zygote polar- isation has been extensively studied in recent years, highlighting the role of the centrosomal kinase Aurora-A (AIR-1) in controlling the cortical distribution of RhoGEF (ECT-2) and actomyosin contractility during polarisation. Although genetic experiments have demonstrated their function in this process, it remains to be tested whether these factors and their interactions are sufficient to induce polarisation.
This *work has provided a theoretical framework to predict the activity of AIR-1 in the cyto- plasm and at the cell cortex, and the cortical distribution of ECT-2 and myosin-II (NMY-2). This framework can recapitulate the dynamic rearrangement of ECT-2 and myosin-II during polarisa- tion, with centrosomes positioned at the posterior pole of the zygote. This model can explain, at least in part, the asymmetric distribution of ECT-2 and myosin-II in the zygote undergoing cytokinesis, suggesting that the mechanism of AIR-1-mediated control of ECT-2 and myosin-II would regulate patterning during polarisation and cytokinesis. This theoretical framework is developed with reason- able assumptions based on previous genetic experiments (except for the myosin-dependent regulation of ECT-2; see comments below).*
Thank you for the positive assessment!

Major issues:

1. *The authors insist that this model correctly predicts the spatio-temporal dynamics of ECT-2 and myosin-II during polarisation and cytokinesis. However, the predicted results do not re- produce the in vivo pattern of ECT-2 in both phases. ECT-2 is cleared from the posterior cortex and establishes a graded pattern across the antero-posterior axis during polarisation (see their previous publication in eLife 2022, 11, e83992, Fig1A -480s) and cytokinesis (see eLife 2022, 11, e83992, Fig1C 60s and 120s). During both stages, ECT-2 does not show local enrichment at the boundary between the anterior and posterior cortical domains in vivo. In fact, when comparing the predicted results with the in vivo pattern of ECT-2 and cortical flow, the authors used non-quantitative descriptions such as 'in good agreement', 'a realistic mag- nitude', 'resemble'. These vague descriptions should be revised and a quantitative assessment of ECT-2 distribution between in silico and in vivo should be included in a revised manuscript.*
As mentioned on p. 1, in the revised manuscript we interacted with the data in a much stronger way. We first used data during pseudo-cleavage to infer the ECT-2/myosin relationship. We then examined (Fig. 3) quantitatively how the ECT-2 accumulation during polarization matches the experimental data (it matches early but not later stages). We repeated this for cytokinesis in Fig. 5, where we compared the ECT-2 profile across four experimental conditions to the model prediction.

2. *I assume that the strange local enrichment of ECT-2 at the anteroposterior boundary is due to their assumption that the binding rate of ECT-2 is increased by a linear increase via cortical myosin-II (page 6). This assumption is not directly supported by experimental evidence. A previous study by the same group (eLife 2022, 11, e83992) showed that a progressive increase in ECT-2 concentration at the anterior cortex is partially accompanied by an increase in cortical flow and transport of myosin-II from the posterior pole to the anterior cortex. This observation supports the idea that ECT-2 may associate with cortical components transported by myosin-II based cortical flow. This unrealistic assumption makes the predicted distribution pattern of ECT-2 almost identical to that of cortical myosin-II, resulting in an increase in the concentration of ECT-2 at the anteroposterior boundary where myosin-II forms pseudo- cleavages and cleavage furrows. The authors should clarify why their mathematical model used this assumption and provide a comprehensive analysis and evaluation of the parameter value for an ECT-2-myosin-II interaction.*

© 2025. Published by The Company of Biologists under the terms of the Creative Commons Attribution License (https://creativecommons.org/licenses/by/4.0/).

In the revised manuscript, we outlined the justification for this assumption after presenting the model equations. In Appendix B.3, we were able to constrain all parameters except the recruitment term. Then, in Appendix B.3.3, we provided an analysis of how polarization changes when the recruitment term is increased. We show that the ECT-2 asymmetries with myosin flows are the same as those simply due to AIR-1 inhibition (since the lifetime of ECT-2 is small). Adding indirect recruitment gives asymmetries that resemble experimental data from early establishment of polarity. We showed this both by assuming "myosin" (a species which colocalizes with myosin) recruits ECT-2 (Fig. S4) and by simulating an alternative model (Eq. (S4)) where an explicit species that is advected with cortical flows recruits myosin (Fig. S7).

Original submission

First decision letter

MS ID#: jcs.264093

MS Title: A minimal mathematical model for polarity establishment and centralspindlin-independent cytokinesis

Authors: Ondrej Maxian; Katrina Longhini-Aldis; Michael Glotzer

Article Type: Review Commons Transfer

Dear Dr Glotzer,

Many thanks for transferring your paper to Journal of Cell Science from Review Commons. Following assessment of the paper and your response to the Review Commons referee reports, I am happy to tell you that your manuscript has been accepted for publication in Journal of Cell Science, pending standard publication integrity checks.

Thank you for sending your manuscript to Journal of Cell Science through Review Commons.

© 2025. Published by The Company of Biologists under the terms of the Creative Commons Attribution License (https://creativecommons.org/licenses/by/4.0/).
